# AR cooperates with SMAD4 to maintain skeletal muscle homeostasis

**DOI:** 10.1007/s00401-022-02428-1

**Published:** 2022-05-06

**Authors:** Mitra Forouhan, Wooi Fang Lim, Laura C. Zanetti-Domingues, Christopher J. Tynan, Thomas C. Roberts, Bilal Malik, Raquel Manzano, Alfina A. Speciale, Ruth Ellerington, Antonio Garcia-Guerra, Pietro Fratta, Gianni Sorarú, Linda Greensmith, Maria Pennuto, Matthew J. A. Wood, Carlo Rinaldi

**Affiliations:** 1 Department of Paediatrics, University of Oxford, South Parks Road, Oxford, OX1 3QX UK; 2 Central Laser Facility, Science and Technology Facilities Council, Rutherford Appleton Laboratory, Didcot, Oxfordshire UK; 3 Department of Neuromuscular Diseases, UCL Queen Square Institute of Neurology, London, UK; 4 Department of Physiology, Anatomy and Genetics, University of Oxford, Oxford, UK; 5 Department of Neurosciences, Neurology Unit, University of Padova, Padova, Italy; 6 Venetian Institute of Molecular Medicine (VIMM), Padova, Italy; 7 Department of Biomedical Sciences, University of Padova, Padova, Italy; 8 MDUK Oxford Neuromuscular Centre, University of Oxford, Oxford, UK

**Keywords:** Muscle atrophy, Androgens, Androgen receptor, TGFβ pathway, SBMA, Transcriptional cooperativity

## Abstract

Androgens and androgen-related molecules exert a plethora of functions across different tissues, mainly through binding to the transcription factor androgen receptor (AR). Despite widespread therapeutic use and misuse of androgens as potent anabolic agents, the molecular mechanisms of this effect on skeletal muscle are currently unknown. Muscle mass in adulthood is mainly regulated by the bone morphogenetic protein (BMP) axis of the transforming growth factor (TGF)-β pathway via recruitment of mothers against decapentaplegic homolog 4 (SMAD4) protein. Here we show that, upon activation, AR forms a transcriptional complex with SMAD4 to orchestrate a muscle hypertrophy programme by modulating SMAD4 chromatin binding dynamics and enhancing its transactivation activity. We challenged this mechanism of action using spinal and bulbar muscular atrophy (SBMA) as a model of study. This adult-onset neuromuscular disease is caused by a polyglutamine expansion (polyQ) in AR and is characterized by progressive muscle weakness and atrophy secondary to a combination of lower motor neuron degeneration and primary muscle atrophy. Here we found that the presence of an elongated polyQ tract impairs AR cooperativity with SMAD4, leading to an inability to mount an effective anti-atrophy gene expression programme in skeletal muscle in response to denervation. Furthermore, adeno-associated virus, serotype 9 (AAV9)-mediated muscle-restricted delivery of BMP7 is able to rescue the muscle atrophy in SBMA mice, supporting the development of treatments able to fine-tune AR-SMAD4 transcriptional cooperativity as a promising target for SBMA and other conditions associated with muscle loss.

**Supplementary Information:**

The online version contains supplementary material available at 10.1007/s00401-022-02428-1.

## Introduction

Skeletal muscle, which accounts for over 40% of the total mass in healthy individuals, plays a central role in maintenance of organismal homeostasis [59]. Conversely, muscle atrophy upon acute and chronic conditions, ranging from genetic muscular dystrophy to critical illnesses, cachexia and sarcopenia, significantly correlates with levels of disability and is an important predictor of mortality [6]. Despite the urgent medical need, treatments able to efficiently counteract muscle loss are lacking due to an incomplete understanding of the underlying intricate molecular mechanisms of regulation. Androgens exert potent anabolic and ergogenic effects on muscle: administration of testosterone or its synthetic analogue oxandrolone result in significant increase in muscle mass secondary to induced protein synthesis [25, 81] and higher testosterone levels are associated with reduced loss of lean body mass in older men [39]. Nevertheless, clinical applications of androgenic therapies are severely limited by the potentially serious side effects associated with chronic administrations, including increased risks of developing cardiomyopathy [5], myocardial infarctions [61], and hypogonadism [16]. Despite these concerns, androgens and anabolic androgenic steroids remain the most widely used doping agents in sport, according to the World Anti-Doping Agency (WADA) (https://www.wada-ama.org/sites/default/files/resources/files/2019_anti-doping_testing_figures_en.pdf). Effects of androgens in both reproductive and non-reproducing tissues, including muscle and brain, are largely mediated by binding to the Androgen Receptor (AR), a ubiquitously expressed transcription factor of the nuclear receptor superfamily. Structurally, the AR comprises an N-terminal domain (NTD), a DNA-binding domain (DBD), a linker or hinge interdomain, and a C-terminal ligand-binding domain (LBD). Agonist interaction with the pockets of AR LBD triggers conformational changes, nuclear translocation, and DNA binding, followed by activation of a wide range of gene expression programmes [21, 27, 64]. Notably, only 7% of AR binding sites display androgen-dependent activation [31] and the AR cistrome is heavily influenced by direct or indirect interaction with other transcription factors, such as FOXA1, HOXB13, and GATA2 [66, 68], which at least partially accounts for the cell-specific effects of AR activation. Here we sought to investigate the molecular mechanisms underpinning AR transcriptional regulation of muscle homeostasis in health and disease, which are currently unknown. As an ideal model of study, we employed spinal and bulbar muscular atrophy (SBMA or Kennedy’s disease, OMIM #313200), a monogenic, adult-onset, neuromuscular condition, caused by a CAG expansion in the *AR* gene [38]. The encoded elongated polyQ stretch confers proteotoxic effects, which cause extensive transcriptional dysregulation, ultimately leading to cell dysfunction and death, primarily by a toxic gain-of-function [60]. Recent evidence has shown that the severe and progressing muscle weakness and atrophy observed in affected individuals results from not only denervation secondary to lower motor neuron degeneration, but also a primary muscle pathology, by mechanisms which are poorly elucidated [48, 83]. Moreover, muscle-restricted genetic correction and treatments only targeting the peripheral tissues are sufficient to rescue the disease phenotype in preclinical models of SBMA and other diseases of the motor unit [10, 15, 42, 65], further supporting skeletal muscle as a major contributor of the pathogenesis and a target for therapy for these conditions.

## Materials and methods

### Human samples

The Neuromuscular Bank of Tissues and DNA samples at the University of Padova, member of the Telethon Network of Genetic Biobanks (project no. GTB12001), funded by Telethon Italy, provided us with the skeletal muscle samples used for the RNA sequencing. Anonymized control and patient sample collection and storage was approved by the local Ethics Committee, as previously described [9]. Briefly, after obtaining written informed consent from each individual, 100–200 mg of muscle tissue was collected using an open biopsy procedure from 9 SBMA patients followed at the Neuromuscular Clinic of the University of Padova (2 quadriceps femoris, 1 triceps brachii, 1 biceps brachii, 5 vastus lateralis) and 4 age- and sex-matched healthy subjects (vastus lateralis), with no signs of neuromuscular diseases and with normal creatine kinase levels. All biopsied muscles from SBMA individuals showed signs of weakness and atrophy. CAG repeat sizes and clinical characteristics are summarized in Supplementary Table 1, online resource.

### Mice

Experiments were performed in the Biomedical Sciences Unit at the University of Oxford, according to procedures authorized by the UK. Home Office (Animal Scientific Procedures Act 1986). Mice were housed in individually ventilated caging systems, with access to food and water *ad libitum*. AR100Q transgenic animals [13] and the Taiwanese Smn^−/−^;SMN2 (RRID: J:59313) mice [30] were kindly provided by the Pennuto and the Wood laboratories, respectively. Only male mice were used in the analyses. Dual promoter AAV vector plasmids containing an expression cassette consisting of the muscle-specific enhanced muscle creatine kinase promoter (Enh358MCK) followed by human BMP7 cDNA or mock sequence and human CMV promoter followed by cDNA-encoding GFP were provided by SignaGen Laboratories (Rockville, MD). A viral load of 2/2.5 × 10^11^ vg was injected into the tail veins of SBMA AR100Q mice. Injection volume was brought to 100 µl with 1 × phosphate-buffered saline (PBS). The body weight and grip strength using hanging wire and strength meter (Bioseb) were recorded weekly. Randomization was performed among littermates, the treatments were administered, and analyses were performed by blinded investigators. Study endpoint was set at a body weight loss of > 20%.

### RNA/cDNA preparation and RT-qPCR

Ten milligrams of muscle tissue were homogenised in 1-thioglycerol homogenisation solution for 2 × 1 min using Precellys tissue homogeniser (Bertin instruments). Total RNA from muscle or cell lines was isolated using Maxwell RSC simply RNA tissue or cells kit and Maxwell RSC instrument Promega according to the manufacturers’ protocol. One microgram of RNA was used for cDNA synthesis with the High-Capacity cDNA Reverse Transcription Kit (Applied Biosystems). RT-qPCRs were set up with fast SYBR green master mix (Applied Biosystems) using 20–50 ng of DNA templates. Each reaction was performed in duplicate. A no template control was used along with other samples to check for contamination. The reactions were run with the default parameters of the Applied Biosystems Step One Plus real-time PCR system (for 96-well format). Primers used for the RT-qPCR experiments are listed in Supplementary Table 2, online resource.

### RNA sequencing analysis pipeline

For human tissue libraries, sequence reads were adapter and quality trimmed with Trim Galore! (v 0.3.1, https://github.com/FelixKrueger/TrimGalore). Quality control analyses were performed on both raw and trimmed reads using FastQC (v 0.11.1, https://www.bioinformatics.babraham.ac.uk/projects/fastqc/) and MultiQC (v 0.7) [22]. Trimmed reads were aligned to the human genome (Ensembl GRCh38) using HISAT2 (v 2.1.0) [37], alignment files processed using Samtools (v1.3) [41], and reads counted in known genes (Homo_sapiens.GRCh38.86.gtf) using the htseq-count function and –m union argument from HTSeq (v0.9.1) [3].

For mouse tissue libraries, sequence reads were adapter and quality trimmed with Trim Galore! (v 0.4.1, https://github.com/FelixKrueger/TrimGalore). Quality control analyses were performed on both raw and trimmed reads using FastQC (v 0.11.7, https://www.bioinformatics.babraham.ac.uk/projects/fastqc/) and MultiQC (v 0.9) [22]. Trimmed reads were aligned to the mouse genome (Ensembl GRCm38) using HISAT2 (v 2.2.0) [37], alignment files processed using Samtools (v1.10) [41], and reads counted in known genes (Mus_musculus.GRCm38.101.gtf) using the htseq-count function and –m union argument from HTSeq (v0.12.4) [3].

Read counts files were combined using Python and differential expression analysis performed using DESeq2 (v.1.22.2) [82]. Differences between groups were tested using the DESeq2 contrast function, and Benjamini-Hochberg-adjusted *P* values reported.

Pathway analysis was performed on differentially expressed genes using the fgsea package (v 1.10.0) [79]. Unless otherwise stated, all analyses were performed using default parameters.

### SDS-PAGE and western blotting

Twenty milligrams of muscle tissue were homogenised in RIPA buffer (150 mM sodium chloride, 0.5% sodium deoxycholate, 0.1% SDS, 1% NP-40, 50 mM Tris, pH 8.0) supplemented with PhosSTOP^TM^ and cOmplete^TM^ protease inhibitor cocktail (Roche) for 2X 5 min using Precellys tissue homogeniser (Bertin instruments). The total protein concentration was assayed using the Pierce bicinchoninic acid protein assay (Thermo Scientific) with a bovine serum albumin (BSA) standard curve according to manufacturer’s protocol. Twenty micrograms of protein were added to 2 × SDS–polyacrylamide gel electrophoresis (SDS-PAGE) buffer [4% (*w*/*v*) SDS, 100 mM Tris-base, 20% (*v*/*v*) glycerol, and 0.008% (*w*/*v*) bromophenol blue] supplemented with PhosSTOP™ and cOmplete™ protease inhibitor cocktail (Roche) and loaded into the precast NUPAGE 10% Bis–Tris gel (Invitrogen). The gel was electroblotted onto a nitrocellulose membrane using Invitrogen Novex XCell SureLock Mini-Cell and XCell II blot module. Membranes were blocked for 1 h at room temperature with 5% BSA in TBS containing 0.1% Tween-20 and 2% (*v*/*v*) serum derived from the same species in which the secondary antibody was produced and incubated overnight with the following primary antibodies at 1:1000 dilution: Smad4 (Cell Signaling, 38,454), P-Smad3 (Cell Signaling, 9520), Smad3 (Cell Signaling, 9523), P-Smad1/5 (Cell Signaling, 13,820), Smad1 (Cell Signaling, 6944), P-Akt (Cell Signaling, 9271), Akt (Cell Signaling, 9272), Vinculin (Sigma-Aldrich, hVIN-1). Membranes were then incubated with an appropriate horseradish peroxidase (HRP)-conjugated secondary antibody in 1:5000 dilution, anti-mouse-HRP–conjugated antibody (Invitrogen 62–6520) or anti-rabbit-HRP-conjugated antibody (Invitrogen 31,460). Protein signals were detected in the presence of Pierce ECL Western blotting substrate (Thermo Fisher Scientific) using LI-COR Odyssey.

Infrared Imaging System. The blots were quantified by densitometry analysis using ImageJ software. The density of each band was calculated relative to a loading control and standardised against a control protein sample on each blot.

### Cell lines and reagents

Human embryonic kidney (HEK) 293 T cells were obtained from the American Type Culture Collection (ATCC) and cultured as described on the ATCC cell culture guide. Immortalized human myoblast cells (MRC CNMD Biobank London, L954/1284 M-I) were maintained in skeletal muscle cell growth medium (PromoCell) supplemented with supplement mix (PromoCell), 10% (*v*/*v*) foetal bovine serum (FBS; Gibco), 1 × GlutaMAX (Gibco), and 1 × antibiotic antimycotic (Gibco). C2C12 cells stably express AR24Q or AR100Q were kindly gifted by the Pennuto laboratory. All cell lines were cultured at 37 °C and 5% CO_2_. Where indicated, cells were treated with DHT (Sigma-Aldrich) and recombinant human BMP7 (Gibco, PHC9544) to final concentrations of 10 nM and 50 ng/ml, respectively. Transient transfection of cells was performed with 1 μg of each DNA construct and lipofectamine 2000 (Thermo Fisher Scientific 11,668,030) in a six-well plate following the manufacturer’s protocol.

### Plasmid construction

To generate different FLAG-tagged SMAD4 deletion constructs, each of SMAD4 functional domains was PCR amplified from full length SMAD4 cDNA into a pcDNA3 mammalian expression vector under the control of CMV promoter with an N-terminal FLAG epitope tag. All plasmids were verified by Sanger sequencing. PGEX-AR constructs were a gift from the Pennuto laboratory. To generate C2C12 AR24Q and AR100Q cells stably express Halo-Smad4, the Smad4 cDNA with a C-terminal Halo epitope tag was PCR-amplified and cloned into the PiggyBac (PB) Transposon vector clone (Stratech, PB511B-1). PB Transposon vector and PB Transposase vector (Stratech, PB210PA-1) were transfected at a ratio of 9:1 into C2C12 AR24Q and AR100Q cells plated on 24-well plates (∼50,000 cells per well). One day after transfection, the cells were trypsinized and distributed by serial dilution into fresh tissue culture wells. Drug selection using 10 µg/mL puromycin was started on day 3 and was continued for 2 weeks until the foci became visible. When testing the effect of induction during stable cell construction, 1 µg/mL doxycycline was present in the media in all stages starting 1 day before transfection.

### Co-immunoprecipitation

HEK293T cells were transiently transfected with 1 μg of each desired DNA constructs using lipofectamine 2000 (Thermo Fisher Scientific) and treated with DHT (Sigma-Aldrich) and/or recombinant human BMP7 (Gibco) to final concentrations of 10 nM and 50 ng/ml, respectively, for 12 h, where indicated. Cells were then lysed and nuclear and cytoplasmic fractions were obtained using NE-PER™ nuclear and cytoplasmic extraction kit following the manufacturer’s protocol (Thermo Fisher Scientific). One microgram of each of the following antibodies, mouse monoclonal anti-AR (Santa Cruz Biotechnology 441, sc-7305), monoclonal anti-mouse IgG1 isotype control antibody (Cell Signaling Technology 5415 s), rabbit polyclonal anti-FLAG (Cell Signaling 2368), or monoclonal anti-rabbit IgG1 isotype control antibody (Cell Signaling Technology 3900) was coupled to protein G magnetic Dynabeads (Invitrogen). Nuclear extracts were incubated overnight at 4 °C with the antibody-conjugated beads. Beads were washed for ten times in 1% PBS with Tween 20, followed by elution in 2 × SDS protein gel loading buffer at 100 °C for 10 min and separation on SDS-PAGE gels and Western blotting.

To map the interaction between AR functional domains and SMAD4 in a cell-free environment, FLAG-tagged SMAD4 and each of the GST-tagged AR (NTD, DBD, LBD) constructs were first linearized, ethanol precipitated, and then translated into proteins using coupled in vitro transcription/translation TNT wheat germ (Promega, L4130) and *E. coli* S30 (Promega, L1030) extract systems following the manufacturer’s protocols, respectively. PGEX-GST construct was used as a control. Equal amounts of FLAG-SMAD4 and each of the GST-AR (NTD, DBD, LBD) or PGEX-GST were mixed together and incubated overnight at 4 °C. The mixtures were then incubated overnight at 4 °C with protein G magnetic Dynabeads (Invitrogen) coupled with 1 μg of monoclonal anti-mouse GST antibody (Sigma, G1160). Beads were washed for 10 times in 1% PBS with Tween 20, followed by elution in 2 × SDS protein gel loading buffer at 100 °C for 10 min and separation on SDS-PAGE gels and Western blotting with a rabbit polyclonal anti-FLAG (Cell Signaling 2368) antibody.

### Immunohistochemistry and immunofluorescence

Quadriceps, tibialis anterior, and gastrocnemius muscles were isolated, dissected and snap-frozen in dry ice-cooled isopentane. Tissues were sectioned at 10 μm thickness and processed for hematoxylin and eosin (H&E) and nicotinamide adenine dinucleotide (NADH) staining as previously described [80]. For immunofluorescence experiments, tissue sections and human myoblasts cells seeded on poly-l-lysine-coated coverslips were fixed with 4% PFA for 10 min at room temperature and then permeabilised with 0.3% Triton X-100. Antigen unmasking for Pax7 and pSmad1/5 was carried out by incubating the tissue sections in citrate buffer pH 6.0, heated to > 85 °C for 10 min followed by three times washes in PBS. Cells and tissue sections were blocked in PBS containing 0.3% Triton X-100 and 5% (*v*/*v*) serum derived from the same species in which the secondary antibody was produced and incubated overnight at 4 °C in 1:100 dilution with anti-pSmad1/5 (Cell Signaling 41D10), anti-Pax7 (Active Motif 39803), anti-AR (Abcam, ab133273), anti-Smad4 (Santa Cruz, sc-7966) and 1:400 dilution of anti-dystrophin (Abcam, ab15277) primary antibodies followed by incubation for 1 h at room temperature with an appropriate Alexa Flour-conjugated secondary antibodies (1:1000, Thermo Fisher Scientific). Cells and tissue sections were washed three times with PBS before mounting with VECTASHIELD antifade mountant with 4ʹ, 6-diamidino-2-phenylindole (DAPI) (Vector Laboratories). Slides were allowed to dry before imaging using the 3D Histech Pannoramic 250 slide scanner (3D Histech, Hungary) at Bioimaging facility, University of Manchester.

### Luciferase report assays

To determine the effect of BMP7 and DHT on SMAD4-dependent transcriptional activity, HEK293T cells were transiently transfected with Lipofectamine 2000 reagent (Invitrogen) in Opti-MEM I-reduced serum medium (Gibco) with 0.5 µg of pGL3 BRE Luciferase (45,126, Addgene), 0.5 µg of pRL-TK (Renilla luciferase control) (Promega), and 0.5 µg of AR plasmids. Following 24 h of transfection, cells were washed and treated with BMP7, DHT or an equal volume ethanol as negative control. Cells were harvested after 24 h and firefly and Renilla luciferase substrates (Dual-Luciferase Reporter Assay, Promega) were added, and luciferase activity was measured using a microplate spectrophotometer (CLARIOstar, BMG LabTech) according to the manufacturer’s protocol. Renilla luciferase activity was used as the internal normalization control.

### Chromatin immunoprecipitation

Chromatin immunoprecipitation (ChIP) for Smad4 was conducted on C2C12 cells stably expressing AR24Q or AR100Q. Untransfected MCF7 cells were included as the negative controls. Cells were treated with DHT and BMP7 as previously outlined. Cells were cross-linked with 1% formaldehyde for 10 min, harvested and lysates were sonicated to an average DNA fragment length of 200–400 bp. The experiment was conducted using 300 μg of chromatin and 14 μg of anti-mouse Smad4 (B-8) (Santa Cruz, sc-7966) or anti-mouse IgG1 isotype control (Cell Signaling Technology, 5415 s) antibodies as described previously [75]. DNA samples were purified and subjected to quantitative real-time PCR with the primers listed in Supplementary Table 2 online resource.

### Single molecule microscopy

Single molecule imaging with was performed using a Zeiss Axio Observer Z1 with a 100 × 1.46NA objective. An iLas^2^ illumination system (Cairn Optics) was used to achieve highly inclined illumination. JF549 was excited with a 561 nm CW laser and fluorescence emission collected between 580 and 620 nm. JF549 images were detected using an Andor Ixon + cooled emCCD at frame rates or either 20 Hz or 4 Hz for 800 frames.

### Single molecule tracking

All single-molecule time series data were analysed using the multidimensional analysis software described previously [69]. Briefly, this software performs frame-by-frame Bayesian segmentation to detect and measure features to sub-pixel precision, then links these features through time to create tracks using a simple proximity-based algorithm. Nuclei were manually segmented in each time series to remove molecules tracked outside of nuclei. These tracks were then further filtered to exclude defocused tracks.

### Diffusion analysis

The Python version of Spot-On [28] was used to fit the jump length distributions of the tracks obtained from 20 Hz datasets to a two-state diffusion kinetics model. The variability of the model parameter fits (bound and free fractions and diffusion constants) was estimated by bootstrap resampling of tracks with replacement (1000 resamples per experimental condition). Spot-on parameters and dataset descriptions are in Supplementary Table 3, online resource.

### Single-track analysis and estimation of bound state residence times

Two state Hidden-Markov Models (HMMs) based on gamma distributions were used to determine the bound and free segments of individual tracks. The HMMs were implemented in Python using Pomegranate [76] and trained on the squared displacements of the tracks obtained from the 4 Hz time series. The Viterbi algorithm was then used to predict the most likely sequence of states that generated each track, given the full model. This in turn allowed measurement of the duration of track sections predicted to be in the bound state (the residence time), the time that each binding event occurred and the frequency of these binding events in the tracks. For quantitative comparison of bound state residence times, aspects of survival analysis were employed. The complement of the cumulative distribution function (1-CDF or CCDF) of the residence time was first calculated before being corrected for photobleaching. Estimates of the photobleaching profile were generated for each experiment by plotting the number of detected fluorescent molecules as a function of time. The expected residence time for each experiment was calculated by integrating each CCDF. Corrected CCDFs were also fit to multi-exponential models and a double exponential model of the formula:

$$(1-\mathrm{CDF})= \alpha {e}^{-t/{\tau }_{1}}+(1-\alpha ){e}^{-t/{\tau }_{2}},$$

was found to best fit the data. The variability in the expected residence times and double-exponential fit parameters were estimated by bootstrap resampling of the residence times with replacement (1000 resamples per experimental condition).

## Single-molecule fluorescence in situ hybridisation

To identify *Id1* nascent and mature RNA, design ready RNAsope *Id1* probe (ACD 312,228) was used and in situ hybridisation performed, according to the manufacturer’s instructions. Briefly, cells were fixed in 4% PFA for 30 min at room temperature, followed by dehydration in increasing concentrations of ethanol. Cells were then rehydrated in decreasing concentrations of ethanol and permeabilised with 0.1% Tween-20 for 10 min. Cells were then incubated for 10 min at room temperature with hydrogen peroxide and treated with protease III diluted 1:15 with 1 × PBS for 10 min at RT. Hybridisation was carried out for 2 h in a humidified chamber at 40 °C. Cells were then washed in 1 × wash buffer for 2 min at room temperature. The signal was then amplified by probe hybridisation to a cascade of signal amplification molecules (AMP1-6) according to the manufacturer’s protocol. Cells were washed with 1 × wash buffer for 2 min at room temperature each time. Following the last wash step, signal was detected by incubating the cells with RED working solution for 10 min in a humidify chamber. Cells were then extensively washed in tap water to remove the unbounded fast red substrates and submerged in 0.02% ammonia water and washed again with tap water. Coverslips were then dried in a 60 °C dry oven for at least 15 min before mounting using VECTASHIELD antifade mountant with 4ʹ, 6-diamidino-2-phenylindole (DAPI) (Vector Laboratories). Slides were allowed to dry before imaging using the 3D Histech Pannoramic 250 slide scanner (3D Histech, Hungary) at Bioimaging facility, University of Manchester. Image quantification was performed using FISH-quant automatic quantification (Mueller, 2013) in MATLAB version 7.10.0 (Natick, Massachusetts: The MathWorks Inc.; 2010).

### Data and material availability

All data needed to evaluate the conclusions in the paper are present in the paper and/or the Supplementary Materials. The RNA-Seq data from wild type and transgenic SBMA mice are available for download from Gene Expression Omnibus (GEO) with accession number GSE185977.

### Statistical analyses

A two-tailed Student’s *t* test and an ANOVA test were used to compare the means between two and three or more groups, respectively. Survival times of SBMA mice were determined by Kaplan–Meier estimation, and comparisons were made with the log-rank test. A two-way ANOVA was conducted to compare the effect of the treatment on weights and grip strength of the animals using treatment as a between-subject factor and time as a within-subject factor. Power analysis was performed using G*Power 3.1.9.2 software [19]. GraphPad Prism version 8 was used to perform the statistical analyses (GraphPad, La Jolla, CA). A *P* value less than 0.05 was set as statistically significant.

## Results

### The BMP signaling axis does not efficiently activate a SMAD4 transcriptional programme in SBMA skeletal muscle

**Fig. 1:**
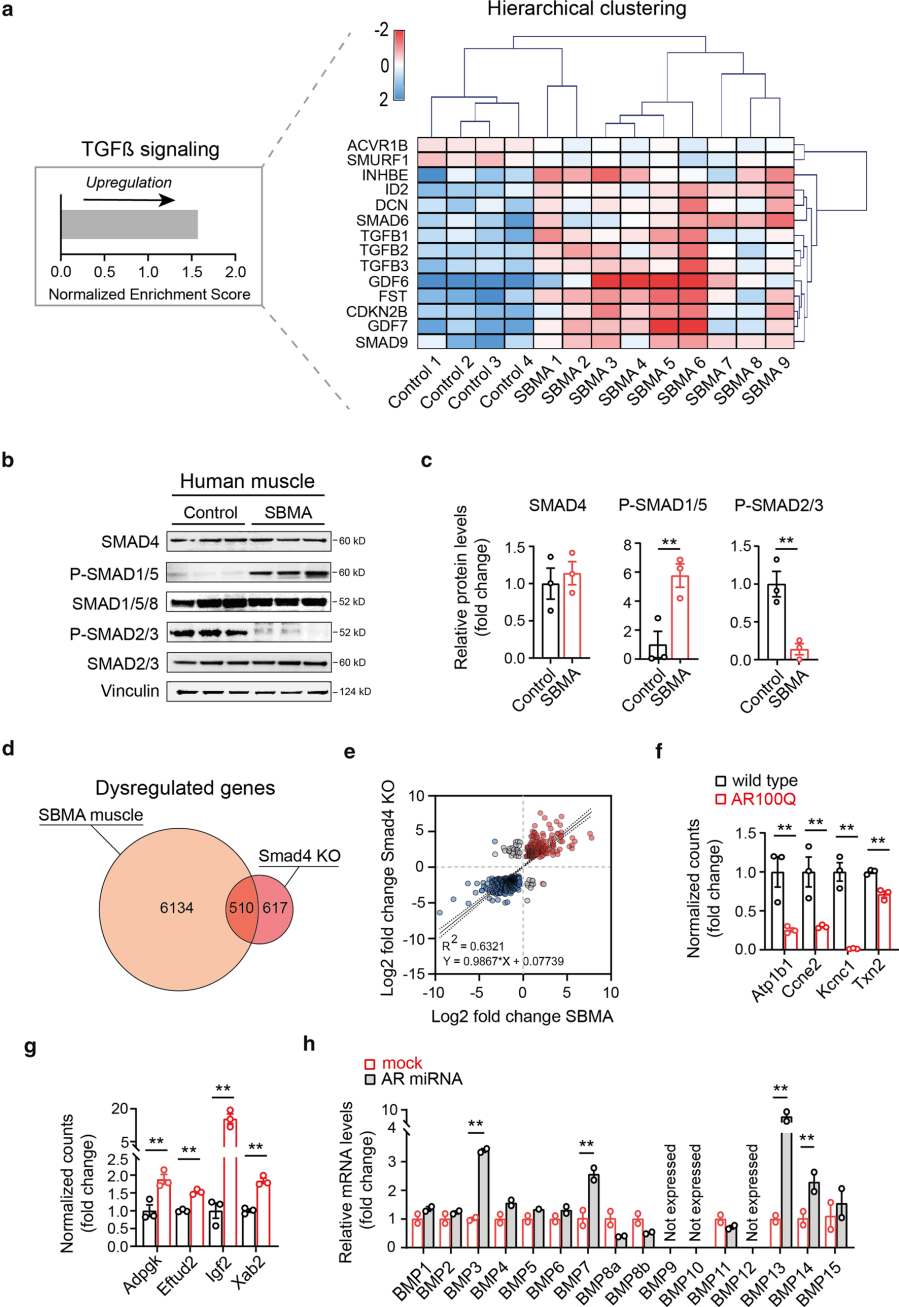
Skeletal muscle in SBMA shows features of functional SMAD4 insufficiency despite BMP pathway activation. **a** KEGG pathway analysis of differentially expressed genes in the transcriptomes of skeletal muscles from SBMA patients and age- and sex- matched unaffected individuals shows that the TGFβ pathway is significantly upregulated in SBMA (left). Normalised counts of genes in the TGFβ pathway from this dataset were used to generate the hierarchical clustering heatmap (right). Multiple Experiment Viewer (MEV) (v 4.8.1) [29] was used to normalise each variable (mean-centring and dividing by the standard deviation) and generate the figure. Upregulated and downregulated genes are displayed in red and blue, respectively. **b** Whole cell extracts from human skeletal muscle of SBMA and age- and sex-matched individuals were resolved by SDS PAGE followed by immunoblotting using SMAD4, P-SMAD1/5, SMAD1/5/8, P-SMAD2/3, and SMAD2/3 antibodies and using Vinculin as loading control (*n* = 3 per group). Size is expressed in kilodaltons (KD) and displayed next to the blot. **c** Quantification of SMAD4, P-SMAD1/5 and P-SMAD2/3 levels relative to the non-phosphorylated SMAD (where applicable) and normalised to Vinculin. Data are mean ± s.e.m. Each dot represents one replicate. **d** Venn diagram illustrates the number of overlapped transcripts between the dysregulated genes in skeletal muscle from SBMA and *Smad4* knock-out (KO) mice upon denervation. **e** Scatterplot for log2-transformed fold changes of the overlapping transcripts, showing significant correlation between skeletal muscle from SBMA and *Smad4* KO mice upon denervation (*R*^2^ and linear function are indicated in the graph). Transcripts upregulated and downregulated in both datasets are indicated in red and blue, respectively. The dashed lines represent 95% confidence interval. **f** mRNA expression levels, expressed as log-normalized counts, in tibialis anterior (TA) muscle of wild type and SBMA mice of genes containing Smad binding elements in their promoter regions and known to be transcriptionally activated and **g** repressed by Smad4 (*n* = 3 per group). **h** mRNA expression levels of BMP genes normalised to *Gapdh* housekeeping gene in TA muscles from 11-week old AR100Q mice upon AR silencing via miRNA compared to mock treated littermates (*n* = 2 per group). Data are mean ± s.e.m. Each dot represents one replicate

To gain insight into the mechanisms of polyQ AR regulation of muscle homeostasis, we employed RNA sequencing of skeletal muscle samples from male SBMA transgenic mice carrying the human AR transgene with pathological glutamine expansion (AR100Q) [13] and wild-type littermates. This transgenic model shows gender-specific, androgen-dependent motor dysfunction, with skeletal muscle pathology characterized by high number of atrophic, angulated and grouped fibers, together with large hypertrophic fibers with central nuclei and modest increase of perimysium connective tissue, overall recapitulating the combined neurogenic and myogenic features observed in skeletal muscle of SBMA patients [83]. Analysis of the differentially expressed genes recently performed by our group [43] revealed that the TGFβ signaling is significantly dysregulated in SBMA muscle. This pathway, together with the AKT-mTOR cascade, with which is highly interconnected, is the main regulator of skeletal muscle homeostasis in adulthood [70]. To confirm the disease relevance of these findings, we performed transcriptomic analysis of skeletal muscles from genetically diagnosed SBMA individuals (*n* = 9; average CAG repeats 46, range 44–50) and age- and sex-matched unaffected controls (*n* = 4) (Supplementary Table 1, online resource). KEGG pathway enrichment studies of this dataset also identified this pathway as dysregulated in SBMA (Fig. 1a), with two genes of the TGFβ cascade being downregulated (*ACVR1B, SMURF1*) and 12 upregulated (*CDKN2B, DCN, FST, GDF6, ID2, INHBE, SMAD6, SMAD9, TGFB1, TGFB2, TGFB3*) (Fig. 1a). This pathway comprises two arms: the activin/myostatin and the BMP axes, which, by phosphorylation of either the SMAD2/3 or SMAD1/5/8 complex, compete over recruitment of the transcription factor SMAD4 to drive a muscle atrophy or hypertrophy transcriptional programme, respectively [70]. Western blot analyses of skeletal muscle samples from SBMA individuals and age- and sex-matched controls showed dramatic increased levels of phosphorylated SMAD1/5 and reduced levels of phosphorylated SMAD2/3 (Fig. 1b, c), suggesting that in SBMA skeletal muscle the BMP axis largely prevails over the competing activin/myostatin axis. Highly concordant results were observed in skeletal muscle of 8-week-old male SBMA transgenic mice, carrying a normal (24Q) or pathological (100Q) polyglutamine stretch in the human AR, which is considered early disease stage in this model [12] (Supplementary Fig. 1a, b, online resource). We next sought to investigate the downstream effects of BMP-SMAD4 activation in skeletal muscle of SBMA patients, and found no change in expression of BMP-negatively regulated *FBXO30* [72], as well as other E3 ubiquitin ligases commonly upregulated in muscle atrophy (*FBXO21, TRIM63*) [7, 51], the master controller of muscle homeostasis *HDAC4* [47] or other atrophy-related genes (*FBXO32, ASB2*), whose induction or repression directly depends on the TGFβ pathway [17, 72, 86], as previously observed [56, 67] (Supplementary Fig. 2, online resource). As mice carrying a *Smad4* deletion exclusively in muscle displayed features of severe primary muscle atrophy with increased protein catabolism and peculiarly no activation of atrophy-related genes when subject to fasting or denervation [72], we hypothesised that activation of the BMP signaling in SBMA fails to generate an anti-atrophic response due to a functional SMAD4 deficiency. Over 45% (510/1127) of the transcripts differentially expressed in skeletal muscle of *Smad4* knock-out (KO) mice upon denervation significantly overlapped with the SBMA mice transcriptomic profile (Fig. 1d), the near totality of which (483/510, 94%) following the same pattern of dysregulation, with 186 transcripts being upregulated (Representation factor: 1.6; *P* < 0.0001) and 297 downregulated (Representation factor: 1.6; *P* < 0.0001) in both data set and to a similar degree of fold change (Fig. 1e). Transcripts known to be activated or repressed by specific binding of the SMAD1/5/8-SMAD4 transcriptional complex to their promoter regions [23] were, respectively, downregulated (Fig. 1f) and upregulated (Fig. 1g) in skeletal muscle of SBMA mice, further supporting a SMAD4 functional deficiency in this disease. Notably, mRNA levels of BMPs 1–15, which are subjected to a SMAD4-dependent positive feedback regulation [18], were largely upregulated in the muscle of spinal muscular atrophy mice, which undergo denervation due to lower motor neuron degeneration [44] (Supplementary Fig. 3, online resource), and of SBMA transgenic mice upon AR silencing in peripheral tissues using a previously described miRNA approach [62] (Fig. 1h), overall suggesting a negative effect of polyQ AR on SMAD4 transcriptional activation in skeletal muscle.

### SMAD4 forms a transcriptional complex with AR upon receptor activation

**Fig. 2:**
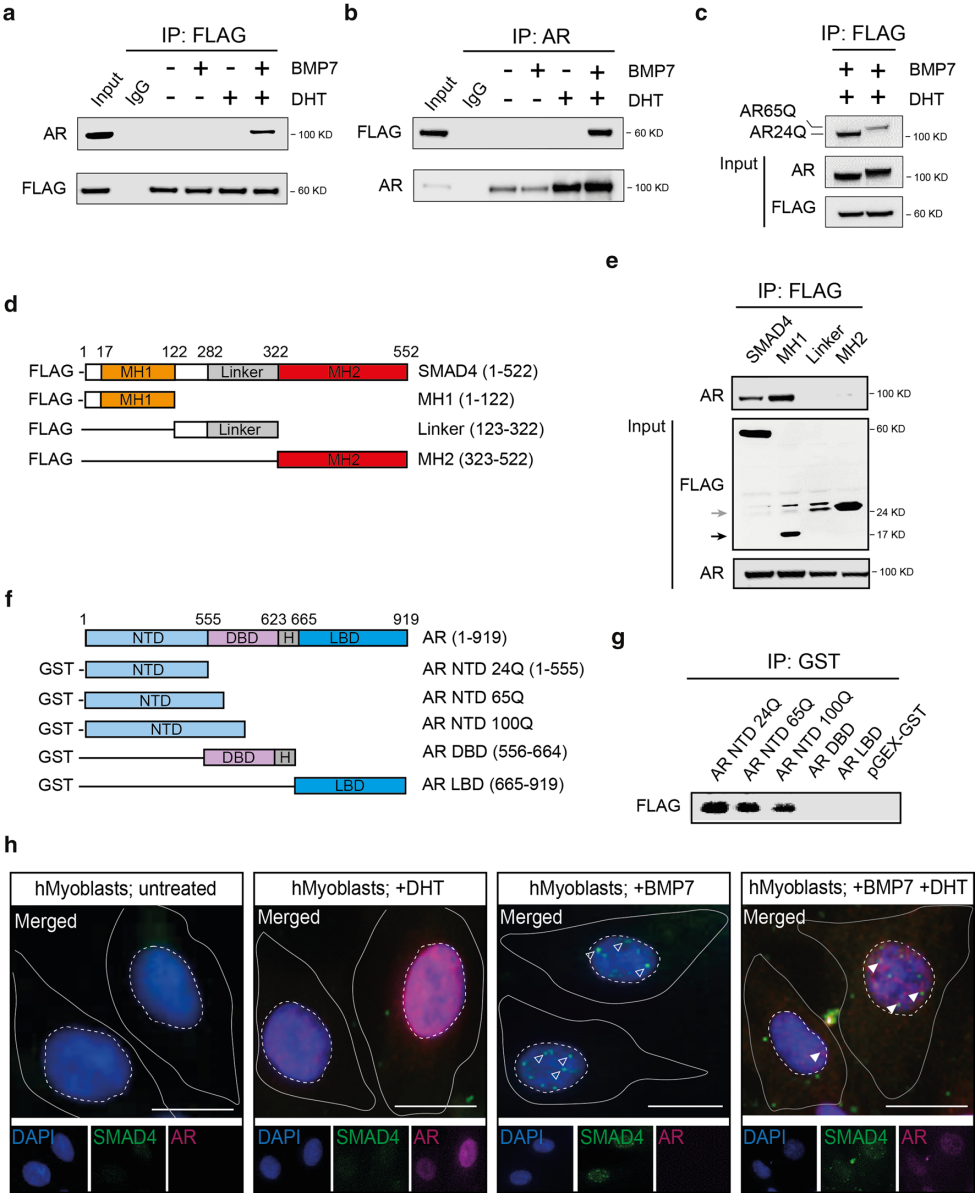
SMAD4 interacts with AR upon BMP7 and DHT induction. **a**, **b** HEK293 were transfected with 1 µg FLAG-tagged SMAD4 and AR24Q constructs treated with DHT 10 nM and BMP7 50 ng/mL for 12 h. Nuclear extracts were fractionated and subjected to immunoprecipitation using an anti-FLAG or **b** anti-AR antibody and anti-IgG as control. Immunoprecipitates were analysed for the presence of AR and SMAD4, **c** HEK293T cells were transfected with 1 µg FLAG-tagged SMAD4 and AR24Q or AR100Q constructs and treated with DHT 10 nM and BMP7 50 ng/mL for 12 h. Nuclear extracts were fractionated and subjected to immunoprecipitation using an anti-FLAG antibody. Immunoprecipitates were analysed for the presence of AR and SMAD4. **d** Schematic of FLAG-tagged SMAD4 constructs used for the co-immunoprecipitation experiment. Amino acid numbers refer to full length SMAD4. *MH1/2* MAD-homology domain 1/2. **e** MH2 domain of SMAD4 interacts with AR. Proteins from HEK293 cells transfected with AR24Q and various FLAG-tagged SMAD4 deletion constructs, treated with DHT 10 nM and BMP7 50 ng/mL for 12 h and subjected to immunoprecipitation with FLAG-beads followed by immunoblotting with anti-AR antibody. Black arrow indicates MH1 domain, grey arrow indicates Linker domain. In blots in **a**–**c** and **e**, 0,1% total lysate was used as input control. Size is expressed in kilodalton (KD) and displayed next to the cropped blots. **f** Schematic of GST-tagged AR constructs. Amino acid numbers refer to full length AR. NTD: N-terminal domain; DBD: DNA binding domain, LBD: ligand binding domain. **g** NTD domain of AR interacts with SMAD4. One microgram FLAG-tagged SMAD4 and GST-tagged AR deletion constructs were translated into proteins in a cell-free environment. Equal amounts of in vitro translated proteins were combined and immunoprecipitated using Halo beads followed by immunoblot analysis with anti-GST antibody. **a**–**c**, **e**, **g** Representative images of 3 biological replicates. **h** Endogenous association of AR and SMAD4 upon DHT and BMP7 treatment in the nucleus of human myoblasts. Representative immunofluorescence micrographs of human myoblasts treated with DHT 10 nM and BMP7 50 ng/mL for 12 h and stained with anti-SMAD4 antibody (green), anti-AR antibody (magenta), and DAPI (*n* > 100 cells from 3 biological replicates). White empty and filled arrowheads indicate SMAD4 and SMAD4-AR signal, respectively. Scale bar, 20 µm

Following the evidence that AR interacts with SMAD proteins in prostate cancer [34], we proposed that SMAD4 dynamically associates with AR to orchestrate specific gene expression programmes in a time- and context-dependent fashion. Immunoprecipitation experiments in HEK293T cells transfected with N-terminal FLAG-tagged SMAD4 and AR vectors showed that such association occurs upon treatment with SMAD4-transcriptional activator BMP7 and dihydrotestosterone (DHT), which induces AR nuclear translocation (Fig. 2a–c). BMP7 was chosen among other BMP ligands because of its well-established role as a positive regulator of muscle mass through the SMAD1/5/8-SMAD4 pathway [2]. We next generated deletion variants harbouring only the MAD homology (MH) 1, 2, or the linker region of SMAD4 (Fig. 2d), and mapped this interaction to the MH1 region (Fig. 2e). This domain, upon activation of the TGFβ receptor, becomes available as a binding platform for other transcription factors and binds to DNA, while the MH2 domain is mainly responsible for the interaction with receptors and oligomer formation with other SMADs [4]. To further map the physical association with AR and determine whether pre-emptive interaction with the DNA template is necessary, we created a series of glutathione S-transferase (GST)-tagged AR constructs for in vitro pull-down studies (Fig. 2f) and demonstrated that the AR NTD, essential for AR transactivation, binds directly to SMAD4 (Fig. 2g). Notably, the polyQ AR maintained the ability to interact with SMAD4, although to a lesser extent with increasing size of the polyglutamine stretch (~ 70% for AR65Q and ~ 50% for AR100Q compared to wild type) (Fig. 2c, g). By immunofluorescence experiments in human myoblasts we observed nuclear co-localization of endogenous SMAD4 and AR following DHT and BMP7 treatment (Fig. 2h), further suggesting a direct, activation-dependent, interaction between the two proteins.

### AR enhances SMAD4 bound fraction and residence time

**Fig. 3:**
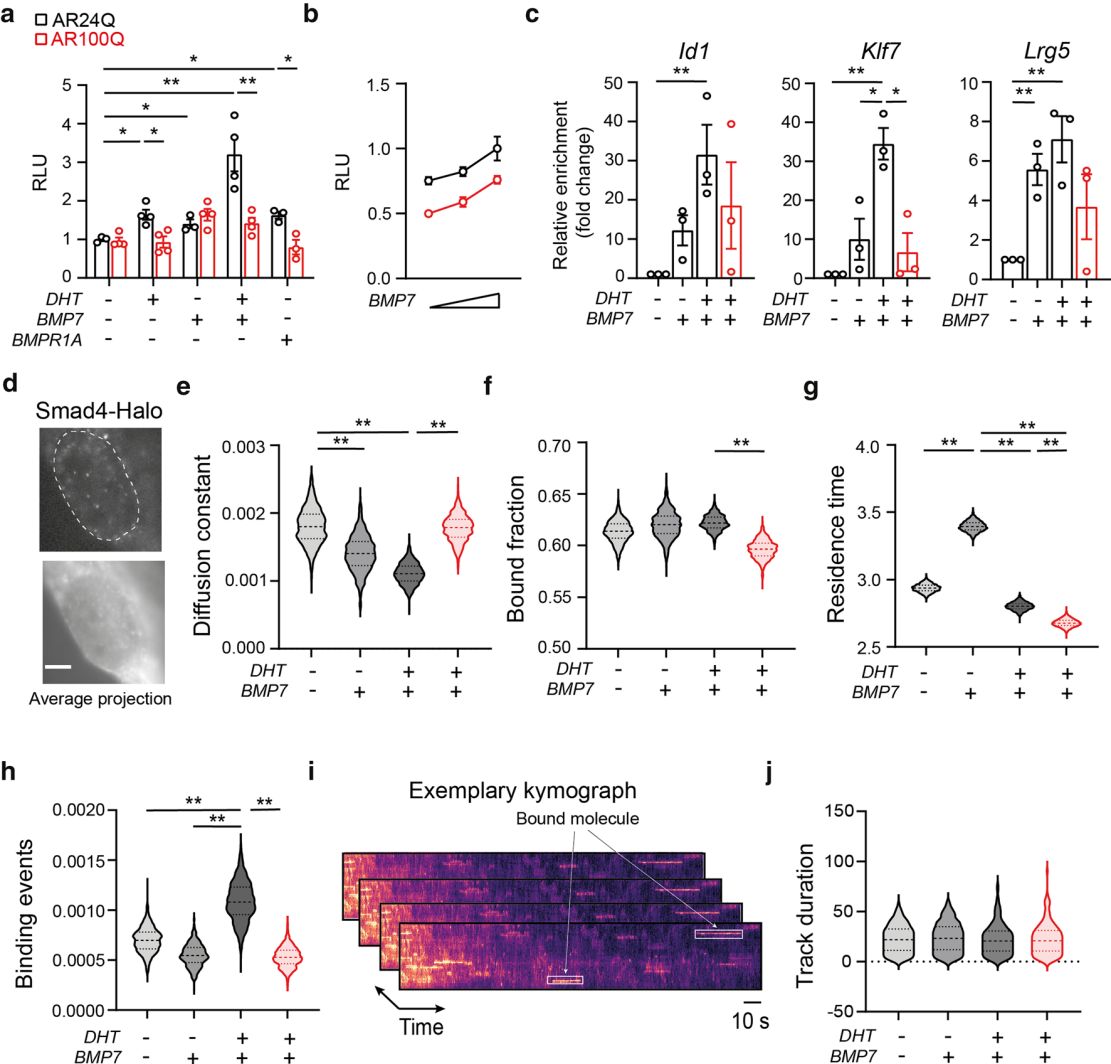
AR modulates SMAD4 DNA binding dynamics. **a** C2C12 cells expressing wild-type (24Q) or expanded (100Q) AR were transfected with the luciferase pARE-E1b-Luc and the *β*-galactosidase pCMVβ reporter constructs, and with a vector expressing a constitutively active BMPR1A harbouring the activating Q233D mutation, where indicated. AR transactivation, expressed as relative luciferase unit (RLU), was measured upon treatment with DHT 10 nM and BMP7 50 ng/mL for 12 h. Data are mean ± s.e.m. Each dot represents one replicate (*n* = 4). **b** AR transactivation was measured as outlined above with increasing concentration of BMP7 (12.5, 25, and 50 ng/mL). **c** Chromatin immunoprecipation assay with anti-Smad4 antibody in C2C12 cells was used to assess direct binding of Smad4 to known binding sites. Data are mean ± s.e.m. Each dot represents one replicate (*n* = 3). **d** Sites of relatively stable immobilization of Smad4-Halo molecules appear as bright spot, allowing the identification of the cell nuclei in C2C12 cells. **e** A two-state diffusion kinetics model was implemented to calculate the estimated diffusion constant and **f** the bound fraction of Smad4-Halo in the bound state of AR24Q and AR100Q expressing C2C12 cells upon treatment with DHT 10 nM and BMP7 50 ng/mL for 12 h. Data are expressed as violin plots, where median and interquartile range are indicated (*n* = 1000 data points per condition). **g** Two state Hidden-Markov Models (HMMs) based on gamma distributions were used to determine the duration of track sections predicted to be in the bound state (the residence time) and **h** the frequency of these binding events in the tracks across the different conditions. **i** Representative kymograph showing chromatin-bound Smad4-Halo (white rectangles) as horizontal segments parallel to the temporal axis. **j** The model provides estimates for the average residence time of Smad4-Halo bound to chromatin upon treatment with DHT 10 nM and BMP7 50 ng/mL for 12 h. Data are expressed as violin plots, where median and interquartile range are indicated (*n* > 500 data points per condition)

We reasoned that AR-SMAD4 transcriptional complex efficiently drives expression of SMAD4 target genes. C2C12 cells stably expressing the human AR transgene with either 24Q or 100Q were transfected with a plasmid consisting of BMP-responsive elements from the *Id1* promoter, fused to a luciferase reporter gene. Significantly increased transactivation was observed upon combined treatment with BMP7 and DHT, compared to BMP7 alone or co-transfection with a plasmid expressing a constitutively active BMP receptor type 1A (BMPR1A) (Fig. 3a), suggesting a cooperative role of AR. This DHT-mediated enhancing effect was lost in the presence of polyQ AR (AR100Q) (Fig. 3a). Increasing BMP7 concentration linearly correlated with the luminescence signal (Fig. 3b). We next investigated SMAD4 promoter occupancy on target genes by chromatin immunoprecipitation (ChIP) assay and found overall increased enrichment upon combined BMP7 and DHT treatment in the context of wild type but not mutant AR (Fig. 3c). To further understand the mobility and binding patterns to chromatin in real-time and at single-molecule resolution, we tracked individual SMAD4 molecules in muscle cells. First, we generated stable AR24Q and AR100Q C2C12 lines expressing SMAD4-Halo, under the control of a Tet-regulated promoter. Halo labelling was chosen as it is well-suited for single-molecule imaging applications [46]. Time-lapse movies at a rate of 4–20 frames per second (fps) allowed the identification of bright spots of SMAD4-Halo signal in the nuclei of C2C12 cells (Fig. 3d). Induction of *Id1* expression upon doxycycline and BM7 treatments was observed, confirming that SMAD4-Halo is transcriptionally active (not shown). The estimated diffusion constant of SMAD4 situated in the nuclei of these cells in a bound state is 0.0018 µm/s^2^, which we note is similar to that reported for chromatin-bound histone H2B (0.0019 µm/s^2^) [50] (Fig. 3e). Treatment with BMP7 led to slower bound state diffusion constant, which was further reduced by addition of DHT, as expected with the increasing crowding density of the transcriptional complex (Fig. 3e). The presence of polyQ AR returned the diffusion constant to around 0.0018 µm/s^2^, hinting at alterations of recruitment of biological molecules for the transcriptional machinery in SBMA (Fig. 3e). No changes were observed in the bound fraction across the different conditions, apart from a slight reduction upon polyQ AR activation (Fig. 3f). For quantitative comparison of bound state residence times, aspects of survival analysis were employed. The complement of the cumulative distribution function (1-CDF) was plotted as a function of time, corrected for photobleaching and fit to multi-exponential models. All datasets fit a double component model with higher precision than a simple exponential model (Supplementary Fig. 4, online resource). A triple exponential model does not improve the fit, suggesting that only two populations of bound molecules account for most of the variability. Expected residence times, as expression of the average time a single molecule is expected to stay in the bound state, were calculated directly from the residence time distributions (Fig. 3g). Decomposition of the bi-exponential fit of the distribution of residence times revealed that the short-lived populations of bound molecules (tau 1: ~ 3 s) largely dominated the residence time distributions over the slow component (tau 2: ~ 8 s) (Supplementary Fig. 5a, online resource). The faster residence times upon DHT treatment suggest more rapid target recognition of the transcriptional complex upon AR activation, rather than increased binding to specific response elements (Fig. 3g; Supplementary Fig. 5a–c, online resource) [11]. To corroborate this observation, we therefore calculated the rate of binding events per nucleus which appeared to be higher upon DHT treatment, suggesting a role of AR in facilitating the search for the cognate sequence (Fig. 3h). Notably, cells with the polyQ AR displayed the shortest residence time and the slowest rate of binding (Fig. 3g,h). The modulation of SMAD4 chromatin-bound fraction can occur upon changes in either its binding time to cognate sites and/or free time between binding events. In order to provide an unsupervised assessment of SMAD4 residence binding times, we analysed kymographs of the single-molecule movies. In this analysis, an immobilized SMAD4 molecule appears as a straight segment parallel to the temporal axis (Fig. 3i). We computed the distribution of SMAD4 track durations and found no difference between the unstimulated and stimulated conditions and with wild-type and polyQ AR (Fig. 3j). Altogether these results indicate that AR facilitates the search process for SMAD4 binding sites rather than modulating the time SMAD4 remains bound on chromatin and that this cooperative function is impaired in SBMA.

### AR activation correlates with SMAD4 transcription

**Fig. 4:**
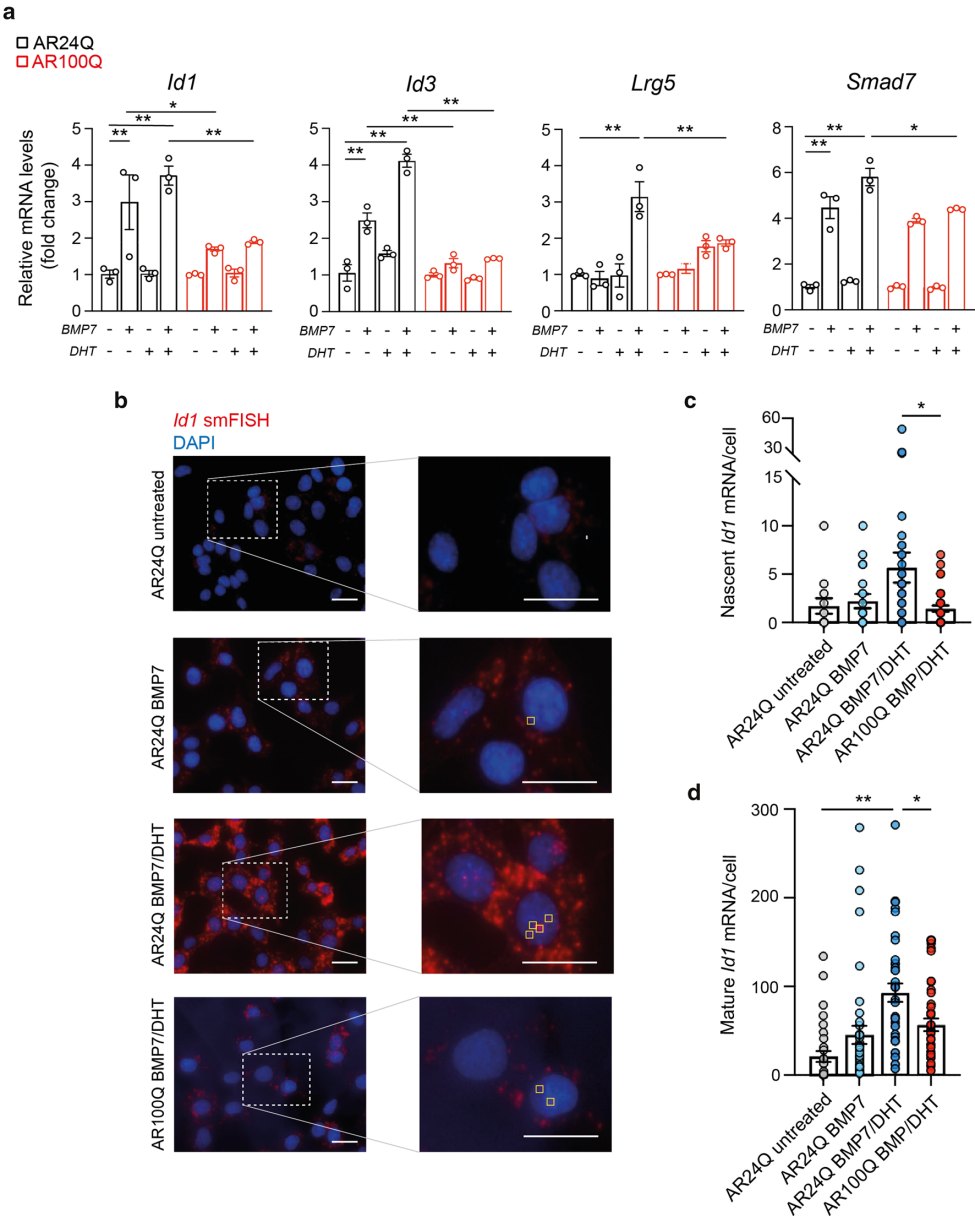
Population and smFISH analyses of SMAD4 targets upon AR transcriptional activation. **a** mRNA expression levels of known Smad4 target genes normalised to *Gapdh* housekeeping gene in C2C12 cells stably expressing AR24Q or AR100Q upon treatment with DHT 10 nM and BMP7 50 ng/mL for 12 h. Data are mean ± s.e.m. Each dot represents one biological replicate (*n* = 3). **b** Representative images of single molecule FISH (smFISH) performed by hybridizing multiple labelled oligonucleotides to *Id1* mRNA (red) in C2C12 cells. Nuclei were stained with DAPI (blue); on the side representative magnification showing nascent RNAs at transcription sites (yellow squares). Scale bar, 20 µm. **c** Average amount of nascent (top panel) and **d** mature (bottom panel) RNA per C2C12 cell. Data are mean ± s.e.m. Each dot represents one measurement from 3 biological replicate (*n* > 10 cells per replicate)

We next tested SMAD4 transactivation ability of known target genes upon treatment with BMP7, DHT, or DHT and BMP7 in mouse C2C12 cells stably expressing human wild type (24Q) or polyQ (100Q) AR transgenes. Increased expression of these target genes upon BMP treatment was further potentiated by DHT, an effect that was not achieved in the presence of polyQ AR (Fig. 4a). Of note, no induction was observed in the selected targets with DHT alone (Fig. 4a).

We further investigated the ability of SMAD4 to induce the expression of *Id1*, a canonical BMP-responsive target gene [45], using single-molecule fluorescence in situ hybridization (smFISH) [24]. This technology allows to distinguish between mature RNA, which appears as individual foci scattered throughout the cell, and nascent RNA, which localises at brighter foci at active transcription sites in the nucleus (Fig. 4b) [40, 55, 78]. In agreement with the bulk RNA assessment (Fig. 4a), DHT and BMP7-treated myoblasts displayed significantly higher amounts of both nascent (Fig. 4c) and mature *Id1* mRNA (Fig. 4d), compared to both unstimulated cells and BMP treated cells only, supporting a transcription-dependent mechanism. This amplified effect was significantly reduced in the presence of polyQ AR (Fig. 4b–d). Overall these results led us to propose a model in which the anabolic activity of the transcription factor SMAD4 in skeletal muscle is enhanced by AR cooperation. Loss of this cooperative effect in SBMA leads to a summed SMAD4 functional transcriptional deficiency, which accounts for the primary muscle atrophy observed in this disease.

### BMP7 delivery overcomes polyQ AR defective cooperation and rescues SBMA muscle atrophy in vivo

We hypothesized that AR cooperates with SMAD4 to drive a muscle hypertrophy programme in vivo and that a malfunction in this enhancing activity conferred by the polyQ tract contributes to the primary muscle atrophy in SBMA.

**Fig. 5:**
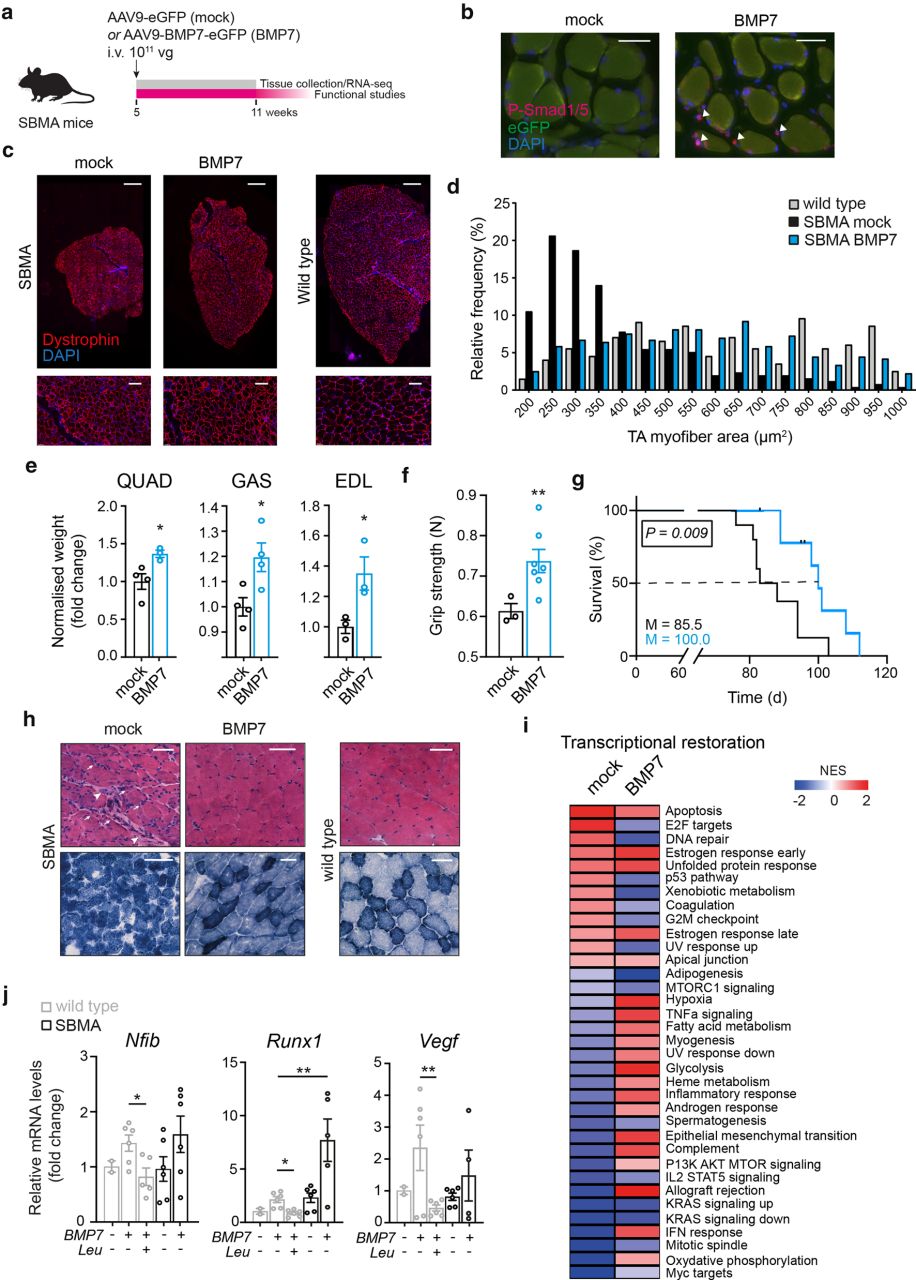
AAV9-mediated delivery of BMP7 in skeletal muscle counteracts muscle atrophy in SBMA. **a** Experimental design; arrow indicates the timing of the intravenous (i.v.) injection. Dose is expressed as vector genome (vg). **b** Representative images of skeletal muscle from SBMA mice treated with AAV9-eGFP (mock) or AAV9-BMP7-eGFP (BMP7), stained with P-Smad1/5 antibody (magenta) and DAPI (blue). Arrowheads indicate the P-Smad1/5 signal. Scale bar, 100 µm. **c** Whole-muscle cross section of TA muscle stained with dystrophin (red) and DAPI (blue) showing partial restoration of muscle atrophy in SBMA muscle upon treatment with BMP7; below representative magnification of myofiber morphology. Scale bar, 1 mm (top) and 100 µm (below). **d** Histogram of distribution of myofiber cross-sectional area in TA muscle of wild type mice and mock and BMP7-treated SBMA mice. **e** Weight of the entire muscle normalised to whole body weight from mock-treated and BMP7-treated SBMA mice. QUAD: quadriceps, GAS: gastrocnemius, EDL: extensor digitorum longus. The colour scheme is conserved across the figure. Data are mean ± s.e.m. Each dot represents one mouse. **f** Grip strength measurements expressed in newtons (N) at week 11 in SBMA mice treated with mock or BMP7 is displayed. Data are mean ± s.e.m. Each dot represents one mouse (*n* = 3 mock-treated and *n* = 7 BMP7-treated SBMA mice). **g** Kaplan–Meier survival estimation of SBMA mice treated with mock or BMP7 (*n* = 10 mice per group) (log-rank test; *M* median). **h** Representative images of H&E (top) and NADH (below) staining of transverse sections of TA muscles from 11-week-old SBMA mice and wild-type littermates. Arrows indicate atrophied myofibers. Arrowheads indicate connective tissue. Scale bars, 50 μm. **i** Heatmap shows the normalized enrichment scores (NES) of gene sets significantly enriched at *P* < 0.05 in the quadriceps muscle of BMP7-treated (BMP7) compared to control SBMA mice (mock), with overexpressed gene sets in red and underexpressed gene sets in blue. Twenty-two pathways out of 35 were partially restored upon BMP7 overexpression in skeletal muscle. **j** mRNA expression levels of known Smad4 target genes normalised to *Gapdh* housekeeping gene in TA muscle from wild-type and SBMA mice upon the indicated treatments. Data are mean ± s.e.m. Each dot represents one mouse (*n* = 2 Leu^−^/BMP7^−^, *n* = 5–6 for the other conditions)

To test the hypothesis that AR cooperative effect on SMAD4 is additive, we generated two adeno-associated viruses (AAVs) expressing human BMP7 cDNA driven by the muscle-specific enhanced muscle creatine kinase promoter (Enh358MCK) and enhanced green fluorescent protein (eGFP) driven by the ubiquitous cytomegalovirus (CMV) promoter (BMP7) or eGFP alone (mock). Male wild type and SBMA transgenic mice carrying the human AR transgene with pathological glutamine expansion (AR100Q) were randomized to receive BMP7-expressing AAV9 or mock by single tail-vein injection at 5 weeks of age, at a dose of 2 × 10^11^ to 2.5 × 10^11^ vector genomes (vg) (Fig. 5a, Supplementary Fig. 6a, online resource). Similar to the patients, this SBMA mouse model shows aberrant BMP activation in skeletal muscle (Supplementary Fig. 1a,b, online resource). Efficient transduction in quadriceps muscle was validated by RT-qPCR, showing increased expression of human BMP7 (Supplementary Fig. 7, online resource), and by immunofluorescence, showing increased phosphorylated SMAD1/5 in the nuclei of myofibers (Fig. 5b), indicative of activation of the BMP pathway. AAV9-mediated delivery of BMP7 induced muscle hypertrophy in wild-type mice, as previously reported [72], an effect that was lost upon chemical castration with Leuprorelin, a luteinizing hormone-releasing hormone (LHRH) agonist that reduces testosterone release (Supplementary Fig. 6b, online resource). BMP7 treatment in SBMA mice promoted near-to-complete normalization of muscle size (Fig. 5c) and cross-sectional myofiber areas (Fig. 5d), with ~ 35% mass increase in quadriceps (QUAD), gastrocnemius (GAS) and extensor digitorum longus (EDL) muscles (Fig. 5e). We observed increased number of Pax7^+^ nuclei and Akt phosphorylation in skeletal muscle of BMP7-treated SBMA mice (Supplementary Fig. 8a–c, online resource), suggesting that the muscle hypertrophy is mediated both by increased proliferation of the satellite cell pool and activation of critical anabolic pathways, as previously demonstrated [26, 84]. Notably, muscle regeneration is not impaired in SBMA muscle [57]. To verify whether BMP7 delivery resulted in functional amelioration, we treated a cohort of SBMA mice using the same treatment paradigm (Fig. 5a). Statistical power analysis was performed on the basis of quantitative measures of body weight, to establish the minimum number of mice required (Cohen’s d effect size: 0.8; minimum sample size for each group: 10 mice). BMP7 treatment resulted in significantly improved end-point grip strength (Fig. 5f), improved rotarod performance (Supplementary Fig. 9, online resource), and increased overall survival (Fig. 5g) in SBMA mice. Histological examination by H&E and NADH staining of tibialis anterior muscles of SBMA transgenic mice showed a dramatic amelioration upon BMP7 treatment of the SBMA pathology (Fig. 5h), which is characterized by a combination of myogenic and neurogenic features with fiber size variability, both round and clustered groups of angulated fibers, central nuclei, mild increase of connective tissue, and a shift from glycolytic to oxidative metabolism, as previously described [48, 67, 83]. To further characterize the effect of BMP7 overexpression in muscle, we next performed transcriptomic analysis in quadriceps muscles from 11-week-old transgenic male mice treated with AAV9-BMP7 or control and wild type littermates. We identified 1204 significantly upregulated and 1103 downregulated genes in SBMA compared to wild type (Supplementary Fig. 10a, online resource). BMP7 treatment resulted in significant restoration of 22/35 SBMA hallmark molecular signature genes (Fig. 5i), with gene expression clusters showing a pattern toward normalization (Supplementary Fig. 10b, online resource). Expectedly, no changes were observed in the levels of denervation-dependent transcripts [1], such as muscle-specific kinase (*Musk*), neural cell adhesion molecule (*Ncam*), and Runt-related transcription factor 1 (*Runx1*), upon muscle-specific BMP7 expression (Supplementary Fig. 11, online resource). BMP induction of representative known SMAD4 targets was blunted in wild type mice treated with Leuprorelin and restored to wild type levels or above in skeletal muscle of BMP7-treated SBMA mice (Fig. 5j), further suggesting that over-activation of the BMP-SMAD4 signaling is able to overcome polyQ AR failed enhancing effect.

## Discussion

The TGFβ pathway plays a prominent role in regulation of muscle mass in adulthood. It comprises over 30 secreted ligands, which, by binding to various tissue-specific combinations of receptor subtypes, orchestrate a wide variety of biological processes, mainly through activation of the SMAD class of proteins [49]. In skeletal muscle, binding of myostatin/growth-differentiation factor (GDF) 8 and GDF11 to the transmembrane activin type IIB and type IIA receptors (ActRIIB/IIA) and TGFβ receptors (TGFβRII) leads to phosphorylation of the SMAD2/3 complex and activation of a muscle atrophy gene expression programme, while binding of the BMP ligands to BMP type II receptor (BMPRII), ActRIIA, and ActRIIB triggers the phosphorylation of the SMAD1/5/8 complex and activation of a muscle hypertrophy programme [70, 74]. Both axes of this cascade impinge on the transcription factor SMAD4, competing over its recruitment, with the BMP being dominant over the myostatin/activin signaling, as *Smad4* knock-out mice exhibit skeletal muscle atrophy and overexpression of the BMP antagonist Noggin is able to revert the hypertrophic phenotype of myostatin-knockout mice [72, 86]. Activation of the BMP signaling is induced in skeletal muscle in response to conditions of increased demand, such as exercise, traumatic nerve injury and denervation [36, 54, 72, 86], to promote satellite cells proliferation [14, 26, 32, 58, 84, 85] and to counteract muscle catabolism by repressing the ubiquitin ligase FBXO30 [72, 86]. Conversely, it has been recently shown that reduction in BMP signaling contributes to the muscle atrophy observed in sarcopenia [77] and cancer cachexia [71].

Here we show that AR forms a transcriptional complex with SMAD4 and enhances its BMP-mediated transcriptional activity to promote skeletal muscle hypertrophy. These results provide the molecular basis for the gender differences in muscle mass and strength [33, 52] and the anabolic effects of androgenic anabolic steroids and androgen-related molecules. Furthermore, testosterone replacement therapy is effective at counteracting muscle atrophy as well as improving quality of life in patients with cachexia [87], suggesting that modulation of the AR-SMAD4 interplay may offer opportunities for treatment. In order to gain further mechanistic insight in disease context, SBMA was employed as a model of study. We showed that the abnormally elongated polyQ tract in AR alters chromatin-binding dynamics and disrupts such enhancing activity on SMAD4 in SBMA, effectively causing a functional SMAD4 deficiency in skeletal muscle. As a consequence, primary muscle atrophy occurs, despite the BMP signaling activation in response to denervation. The expression of atrophy-related genes, including ubiquitin ligases *FBXO21*, *FBXO30*, *FBXO32* and autophagy-related genes such as *CTSL, BECN1, BNIP3*, is not altered in muscle of SBMA individuals, resembling the molecular signature observed in denervated *Smad4* knock-out and Noggin-overexpressing muscles [72], and supporting previous observations that skeletal muscle atrophy in SBMA has distinctive features compared to other forms of atrophy [56, 57, 67]. Intriguingly, our findings suggest that AR modulatory effects are additive and increased activation of the BMP signaling is able to partially overcome the reduced SMAD4 transactivation in SBMA. A predominant myogenic component of the skeletal muscle atrophy in the SBMA transgenic mice is partially to account for the dramatic phenotypic rescue observed upon muscle-specific BMP7 overexpression in this preclinical model. Nath et al*.* have recently reported that diminished function of the transcriptional regulator Myocyte Enhancer Factor 2 (MEF2) is a significant contributor of skeletal muscle atrophy in diseases caused by toxic polyglutamine proteins [56]. SMAD proteins have been shown to physically interact with MEF2 and function as transcriptional co-modulators for MEF2 regulatory proteins [63]. We therefore propose that MEF2 is part of the AR-SMAD4 transcriptional complex, and is subject to the same androgen-mediated enhancing effect to regulate muscle mass.

Whether AR activation leads to changes of the SMAD4 cistrome and why polyQ AR results in lack of transcriptional cooperativity remain to be established. We postulate that changes in AR secondary structure [20] and strength of protein–protein interaction with transcriptional co-regulators [73] upon increasing lengths of the polyQ tract, lead to abnormal formation of phase-separated condensates [8], ultimately resulting in tissue-specific transcriptional dysregulation and the development of clinical phenotypes. As AR-regulated enhancers act as a regulatory hub that increases the likelihood of interactions with other binding sites and transcriptional partners [31], three-dimensional genomic profiling, integrated with ChIP-seq and RNA-seq analyses both in health and disease are necessary to have a comprehensive understanding of AR functions across different cell types and these experiments are currently ongoing in our laboratory. Transcriptional downregulation of TGFβRII is associated with polyglutamine-induced motor neuron damage [35]; therefore, it is conceivable that polyQ AR altered cooperativity with the TGFβ pathway has pathogenic relevance also for tissues other than skeletal muscle. Overall, here we demonstrate a critical mechanism of androgen-mediated regulation of muscle homeostasis. As both AR silencing and excessive activation are associated with toxicity [53, 60], this study supports the development of treatments able to fine-tune AR-SMAD4 transcriptional cooperativity as a promising target for SBMA, cachexia and other conditions associated with muscle loss.

## Supplementary Information

Below is the link to the electronic supplementary material.

Supplementary file1 (DOCX 28 KB)

Supplementary file2 (DOCX 38 KB)

Supplementary file3 (DOCX 32 KB)

Supplementary file4 (DOCX 2378 KB)
