# Supplementary material for: AR cooperates with SMAD4 to maintain skeletal muscle homeostasis
Source: Acta Neuropathol. 2022 May 6;143(6):713–31. doi: 10.1007/s00401-022-02428-1 (PMC9107400; doi:10.1007/s00401-022-02428-1)
Supplement: Supplementary file 1 — Supplementary file1 (DOCX 28 KB) [file 401_2022_2428_MOESM1_ESM.docx]

**SUPPLEMENTARY TABLE 1**

Clinical characteristics

| Sample | Sex | Muscle | Gene | CAG expansion | Age | Age of onset |
| --- | --- | --- | --- | --- | --- | --- |
| Ctrl1 | M | vastus lateralis |  |  | 41 |  |
| Ctrl2 | M | vastus lateralis |  |  | 47 |  |
| Ctrl3 | M | vastus lateralis |  |  | 46 |  |
| Ctrl4 | M | vastus lateralis |  |  | 51 |  |
| SBMA_1 | M | quadriceps femoris | *AR* | 44 | 70 | 60 |
| SBMA_2 | M | quadriceps femoris | *AR* | 45 | 75 | 68 |
| SBMA_3 | M | triceps brachii | *AR* | 46 | 71 | 45 |
| SBMA_4 | M | triceps brachii | *AR* | 46 | 57 | 42 |
| SBMA_5 | M | vastus lateralis | *AR* | 47 | 58 | 41 |
| SBMA_6 | M | vastus lateralis | *AR* | 44 | 84 | 68 |
| SBMA_7 | M | vastus lateralis | *AR* | 49 | 63 | 45 |
| SBMA_8 | M | vastus medialis | *AR* | 45 | 64 | 49 |
| SBMA_9 | M | vastus lateralis | *AR* | 50 | 44 | 35 |
