# Supplementary material for: AR cooperates with SMAD4 to maintain skeletal muscle homeostasis
Source: Acta Neuropathol. 2022 May 6;143(6):713–31. doi: 10.1007/s00401-022-02428-1 (PMC9107400; doi:10.1007/s00401-022-02428-1)
Supplement: Supplementary file 2 — Supplementary file2 (DOCX 38 KB) [file 401_2022_2428_MOESM2_ESM.docx]

**SUPPLEMENTARY TABLE 2**

Primers used for ChIP-qPCR

| Id1 F.P | 5’-AGCCCGTCCGGGTTTTATG-3’ |
| --- | --- |
| Id1 R.P | 5’-TGTGTCAGCGTCTGAACAAG-3’ |
| KIF7 F.P | 5’-ACACTTGCGCTGGCTCCT-3’ |
| KIF7 R.P | 5’-CTCGGATCTGCACTGGGTA-3’ |
| Lgr5 F.P | 5’-TGGCAACCACAAACCCCCGA-3’ |
| Lgr5 R.P | 5’-TTACAATGTGGCCGGCGGCA-3‘ |

Primers used for RT-qPCR

| Id1 F.P | 5’-AGGTGGTACTTGGTCTGTCG-3’ |
| --- | --- |
| Id1 R.P | 5’-CTTGCTCACTTTGCGGTTCT-3’ |
| Id3 F.P | 5’-TCAGACCAGCCTAAGGAAGC-3’ |
| Id3 R.P | 5’-CAGCGGCCGACTCTTATAGA-3’ |
| Smad7 F.P | 5’-TGTGCAAAGTGTTCAGGTGG-3’ |
| Smad7 R.P | 5’-GCCTGCAGTTGGTTTGAGAA-3’ |
| Lgr5 F.P | 5’-GATGCTGCTCAGGGTGGA-3’ |
| Lgr5 R.P | 5’-CGGGTAGCTGACTGATGTTG-3’ |
| Nfib F.P | 5’-AGCCACACCACATCACAGTA-3’ |
| Nfib R.P | 5’-GGATCACTGTGGCTTGGACT-3’ |
| Runx1 F.P | 5’-AGGAAACGATGGCTTCAGAC-3’ |
| Runx1 R.P | 5’-CTCGCTCATCTTGCCGGG-3’ |
| Vegf F.P | 5’-CACGACAGAAGGAGAGCAGA-3’ |
| Vegf R.P | 5’-CACAGGACGGCTTGAAGATG-3’ |
| BMP1 F.P | 5’-GGAGTCCTTGGGAGAGACCTAT-3’ |
| BMP1 R.P | 5’- ACCTCATACTTGGGAACAATGG-3’ |
| BMP2 F.P | 5’-GCAGCTTCCATCACGAAGA-3’ |
| BMP2 R.P | 5’- GATGTGAGAAACTCGTCACTGG-3’ |
| BMP3 F.P | 5’-CGAATGGATTATCTCTCCCAAG -3’ |
| BMP3 R.P | 5’-AACAAGATGCTGAGTGAGGACA-3’ |
| BMP4 F.P | 5’-GACTTCGAGGCGACACTTCTAC-3’ |
| BMP4 R.P | 5’-CAGATGTTCTTCGTGATGGAAA-3’ |
| BMP5 F.P | 5’-AGGAATACACAAACAGGGATGC-3’ |
| BMP5 R.P | 5’-CCAGCAGATTTTACATTGATGC-3’ |
| BMP6 F.P | 5’-TCTTCAGACTACAACGGCAGTG-3’ |
| BMP6 R.P | 5’-ATCACAGTAGTTGGCAGCGTAG-3’ |
| BMP7 F.P | 5’-AGCTTCGTCAACCTAGTGGAAC-3’ |
| BMP7 R.P | 5’-CTGGAGCACCTGATAGACTGTG -3’ |
| BMP8a F.P | 5’-CCTATTACTGTGAGGGGGAGTG-3’ |
| BMP8a R.P | 5’-TGACATTGTTGCTGCTGTCATA-3’ |
| BMP8b F.P | 5’-TCCACTTTGACCTAACCCAGAT-3’ |
| BMP8b R.P | 5’-GTCAGACTCCCTGTTGGAGTG-3’ |
| BMP9 F.P | 5’-CAGATACACAACGGACAAATCG -3’ |
| BMP9 R.P | 5’-CATCATTTTGGCAGGAGACATA-3’ |
| BMP10 F.P | 5’-CTCTACAACAAATTCGCCACAG -3’ |
| BMP10 R.P | 5’-TTCATGGTGAGGGATAGACACA -3’ |
| BMP11 F.P | 5’-CTTGGAAGAGGACGAGTACCAC-3’ |
| BMP11 R.P | 5’-CTGAAGTGGAAATGACAGCAGA-3’ |
| BMP12 F.P | 5’-CATGATGTCGCTTTACAGGAG-3’ |
| BMP12 R.P | 5’-GATACGTCGAACAGGAAGCTCT-3’ |
| BMP13 F.P | 5’-AAGACTTACTCCATTGCCGAGA-3’ |
| BMP13 R.P | 5’-TCGTCCAGTCCTCTGTCTACAA-3’ |
| BMP14 F.P | 5’-ATGCTGACAGAAAGGGAGGTAA-3’ |
| BMP14 R.P | 5’-GCACTGATGTCAAACACGTACC-3’ |
| BMP15 F.P | 5’-GAAAATGGTGAGGCTGGTAAAG-3’ |
| BMP15 R.P | 5’-TCGTATGCTACCTGGTTTGATG-3’ |
