# Supplementary material for: AR cooperates with SMAD4 to maintain skeletal muscle homeostasis
Source: Acta Neuropathol. 2022 May 6;143(6):713–31. doi: 10.1007/s00401-022-02428-1 (PMC9107400; doi:10.1007/s00401-022-02428-1)
Supplement: Supplementary file 3 — Supplementary file3 (DOCX 32 KB) [file 401_2022_2428_MOESM3_ESM.docx]

**SUPPLEMENTARY TABLE 3**

**Spot-On Parameters and Dataset Descriptions**

Spot-On version: Offline Python implementation (FastSPT version 16.1).

Spot-On parameters: states = 2, dZ = 0.7µm, BinWidth = 0.01 µm, MaxJump = 1.25 µm, localisation error fit from data, number of iterations = 3, CDF fit.

Bounds used for fitting algorithm:

D_bound_ = [0.0001, 0.005] µm/s

D_free_ = [0.005, 25] µm/s

F_bound_ = [0, 1]

**Dataset descriptions**

|  | **AR24Q** | **AR24Q**  **+ BMP** | **AR24Q**  **+ BMP + DHT** | **AR100Q**  **+ BMP + DHT** |
| --- | --- | --- | --- | --- |
| Exposure time (s) | 0.05 | 0.05 | 0.05 | 0.05 |
| Number of localisations | 59058 | 40869 | 117744 | 67685 |
| Number of trajectories of length > 3 frames | 5795 | 3265 | 9771 | 4432 |
| Mean trajectory length | 9.5 frames | 11.4 frames | 10.7 frames | 14.4 frames |
| Localization error (µm) | 0.04457 | 0.03905 | 0.04118 | 0.03867 |
| D_bound_ (µm/s^2^) | 0.001807 | 0.001397 | 0.001114 | 0.001775 |
| D_free_ (µm/s^2^) | 0.08283 | 0.0591 | 0.06474 | 0.08605 |
| F_bound_ | 0.6136 | 0.62 | 0.6217 | 0.5964 |
