# Supplementary material for: AR cooperates with SMAD4 to maintain skeletal muscle homeostasis
Source: Acta Neuropathol. 2022 May 6;143(6):713–31. doi: 10.1007/s00401-022-02428-1 (PMC9107400; doi:10.1007/s00401-022-02428-1)
Supplement: Supplementary file 4 — Supplementary file4 (DOCX 2378 KB) [file 401_2022_2428_MOESM4_ESM.docx]

**SUPPLEMENTARY FIGURES**

**AR cooperates with SMAD4 to maintain skeletal muscle homeostasis**

Mitra Forouhan, Wooi Fang Lim, Laura C Zanetti-Domingues, Christopher J Tynan, Thomas C Roberts, Bilal Malik, Raquel Manzano, Alfina A Speciale, Ruth Ellerington, Antonio Garcia-Guerra, Pietro Fratta, Gianni Sorarú, Linda Greensmith, Maria Pennuto, Matthew JA Wood, Carlo Rinaldi

Corresponding author’s contact information:

Carlo Rinaldi, Department of Paediatrics, University of Oxford, South Parks Road, OX1 3QX, Oxford, UK | Phone: +44 (0)1865 272148 | Email:carlo.rinaldi@paediatrics.ox.ac.uk


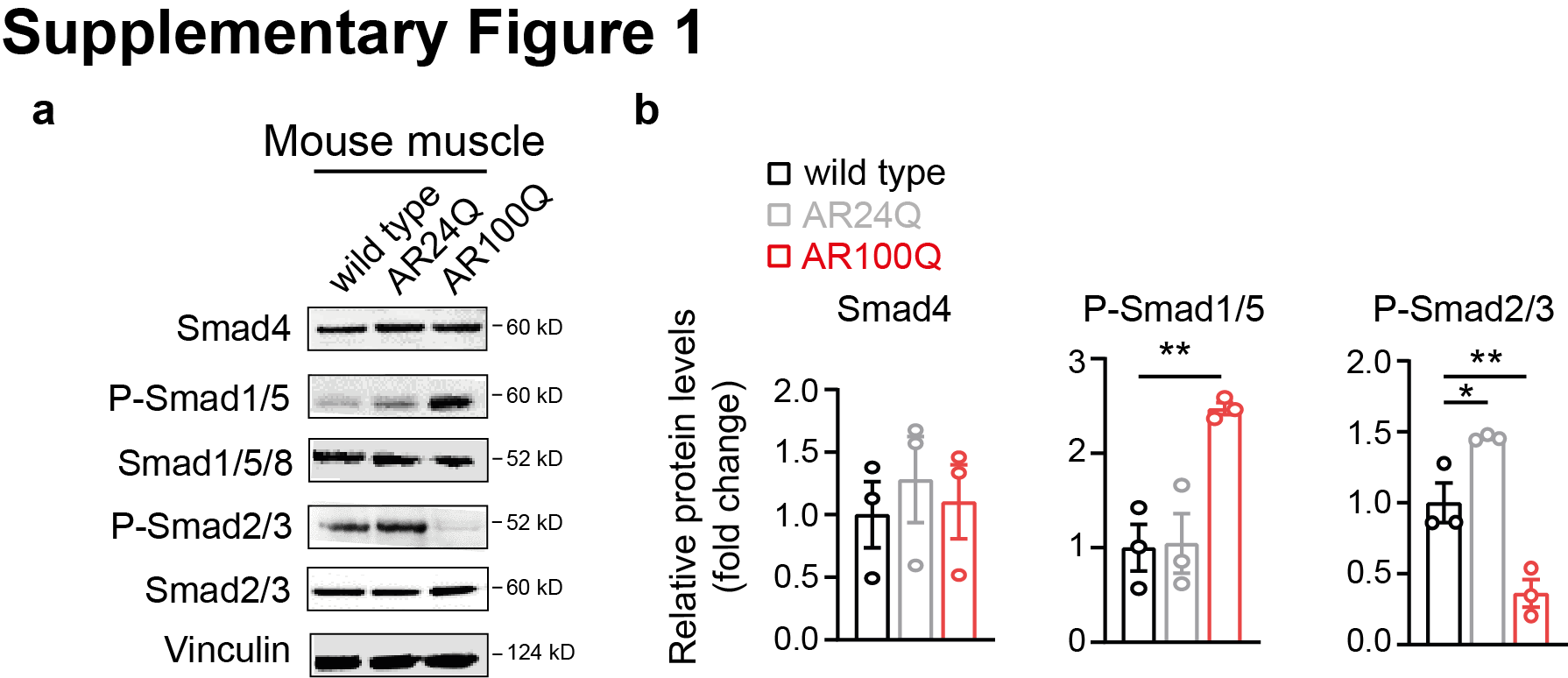


**Supplementary Figure 1. a**, Whole cell extracts from skeletal muscle of 8-weeks old male AR24Q, AR100Q SBMA and wild type mice were resolved by SDS PAGE followed by immunoblotting using Smad4, P-Smad1/5, Smad1/5/8, P-Smad2/3, and Smad2/3 antibodies and using Vinculin as loading control (*n* = 3 per group). Size is expressed in kilodaltons (KD) and displayed next to the blot. **b**, Quantification of Smad4, P-Smad1/5 and P-Smad2/3 levels relative to the non-phosphorylated Smad (where applicable) and normalised to Vinculin. Data are mean ± s.e.m. Each dot represents one replicate.


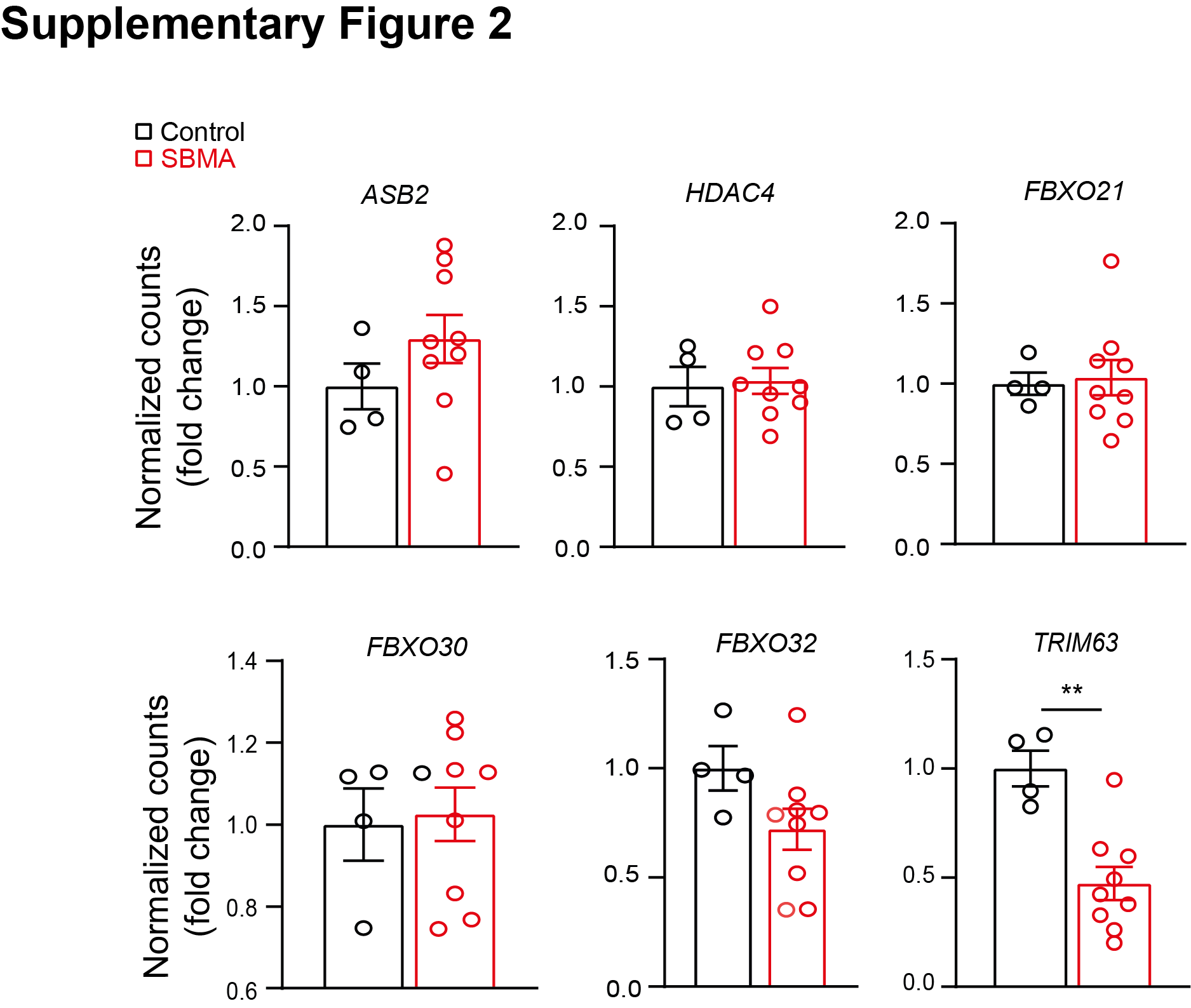


**Supplementary Figure 2.** mRNA expression levels, expressed as log-normalized counts, of genes involved in muscle atrophy in the RNA-seq dataset from skeletal muscle of SBMA (*n* = 9) compared to unaffected individuals (*n* = 4).


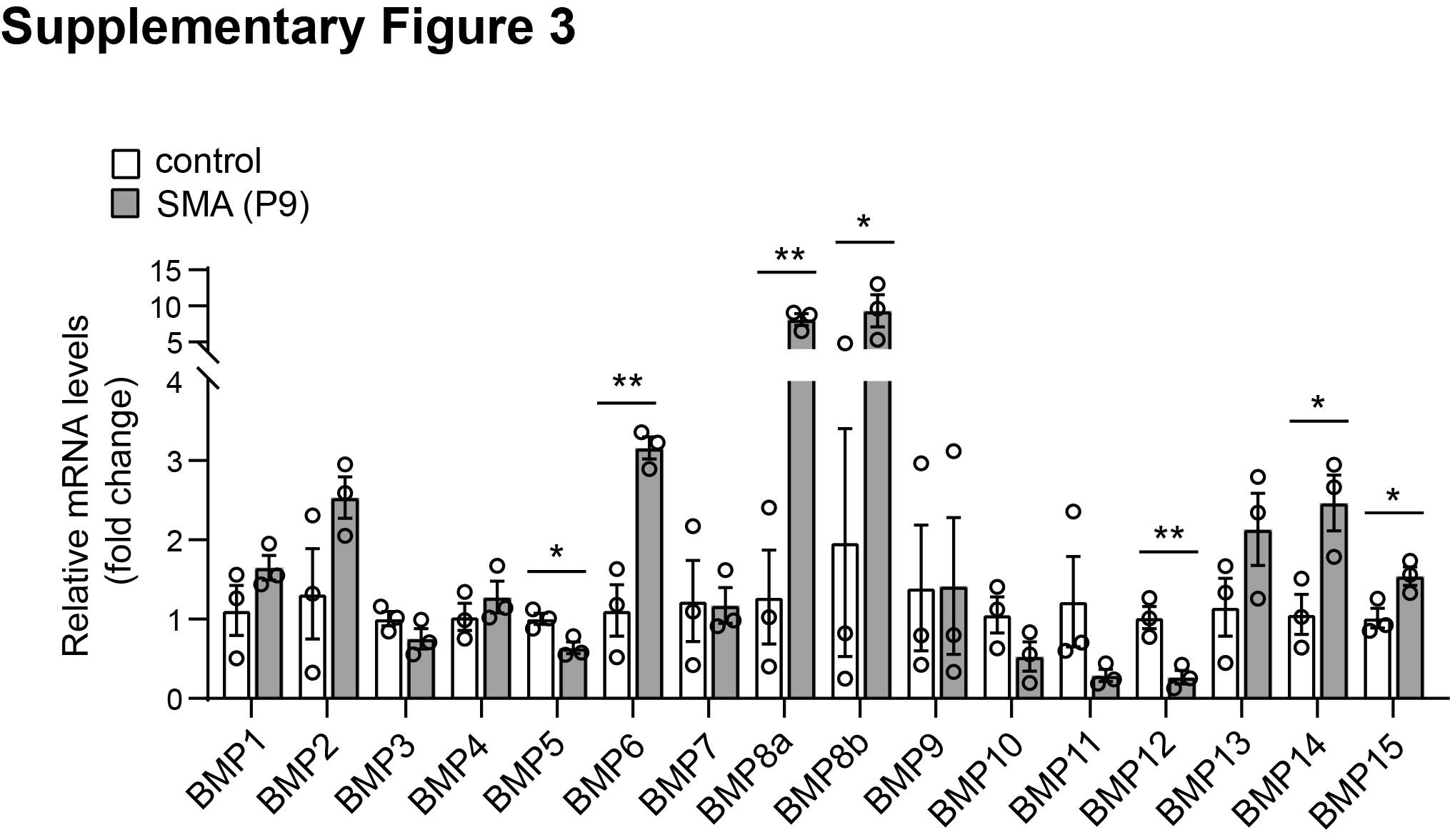


**Supplementary Figure 3.** mRNA expression levels of *BMP* genes normalised to *Gapdh* housekeeping gene in limb muscles from SMA mice at post-natal day 9 (P9) compared to heterozygote littermates (*n* = 3 per group). Data are mean ± s.e.m. Each dot represents one replicate.


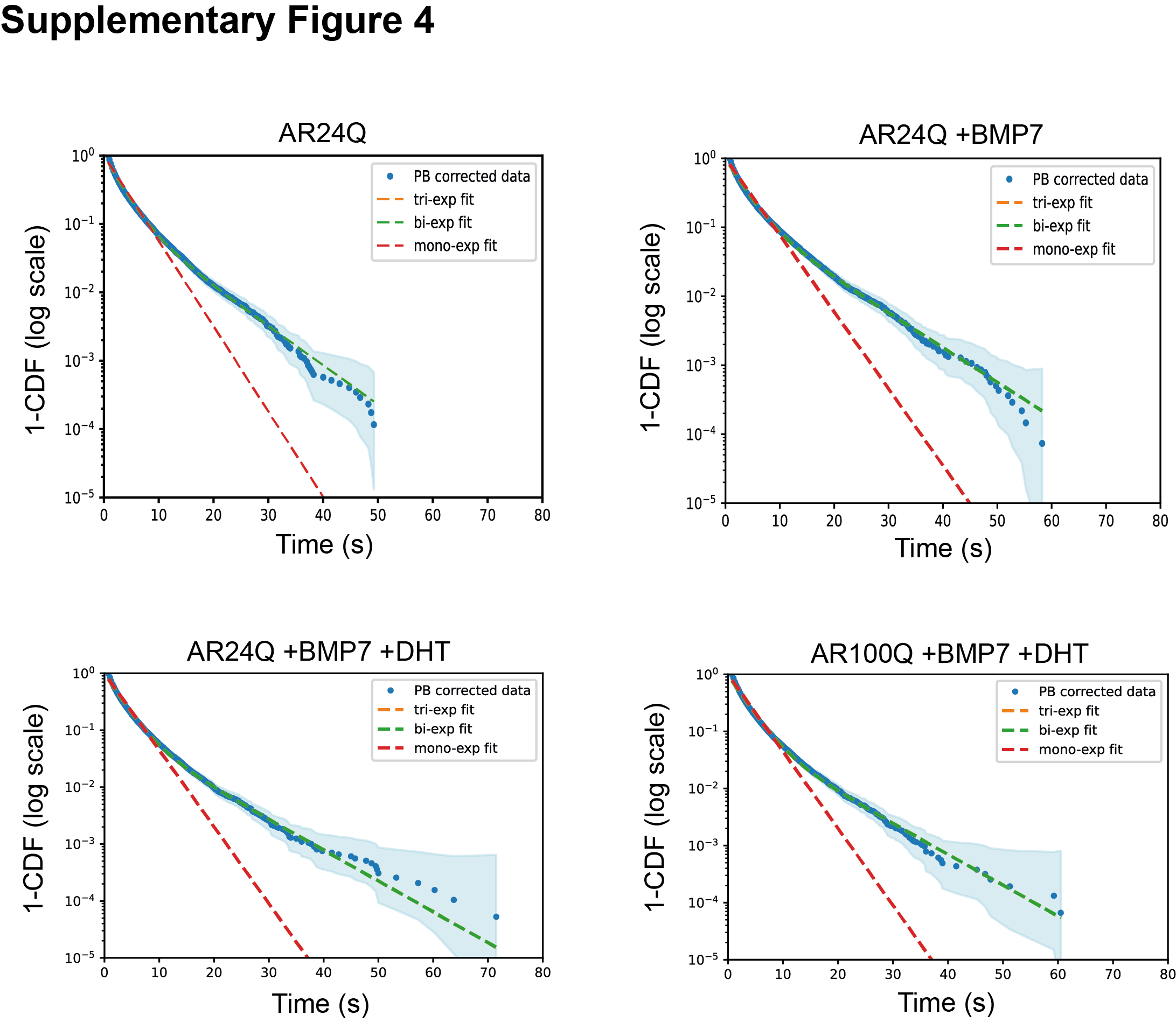


**Supplementary Figure 4.** The complement of the cumulative distribution function (1-CDF) was plotted as a function of time, corrected for photobleaching and fit to multi-exponential models. A double exponential model was found to best fit the data. The shaded region represents the upper and lower bounds of the 95% confidence interval.


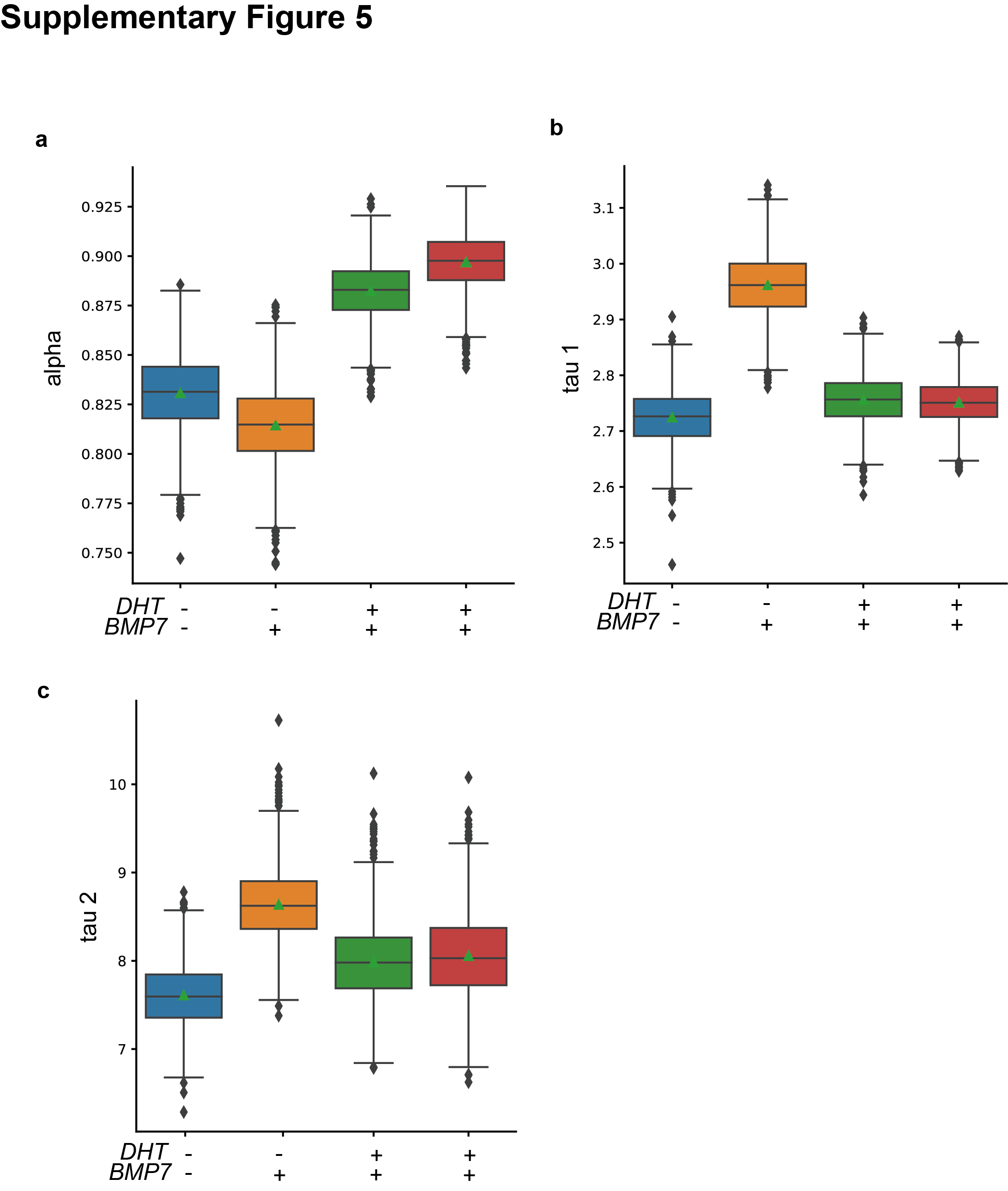


**Supplementary Figure 5. a**, Fractional contribution of the short (~3 s) and long (~8 s) lifetime distributions in C2C12 AR24Q and AR100Q C2C12 cells expressing Smad4-Halo upon DHT 10 nM and BMP7 50 ng/mL for 12 hours, showing that the fast component dominates the residence time distribution. **b**,**c**, Decomposition of the exponential fits, expressed as tau 1 and tau 2 (1-alpha), is displayed. Data are expressed as box plots, where median and interquartile range are indicated (n = 1000 data points per condition).


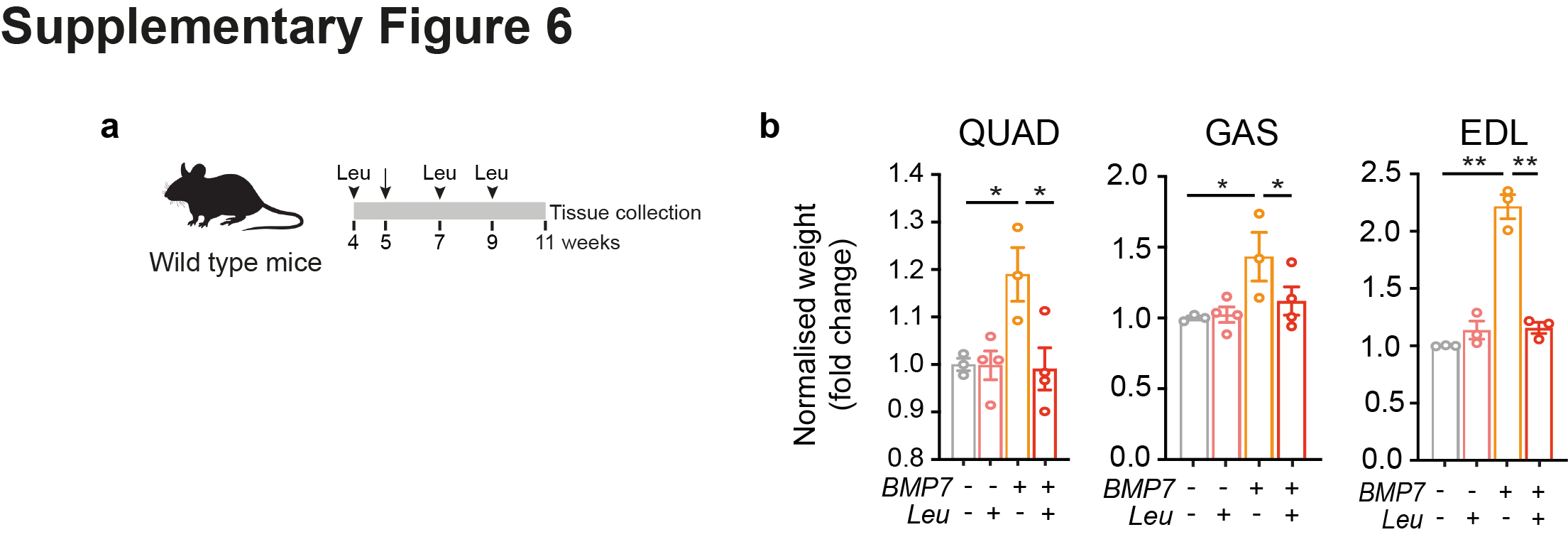


**Supplementary Figure 6. a,** Experimental design; arrow indicates the timing of the AAV9 intravenous injection, arrowheads indicate the timing of the leuprorelin acetate (Leu) subcutaneous injections at a dose of 100 µg per administration. **b**, Weight of the entire muscle normalised to whole body weight from wild type mice upon the indicated treatments. QUAD: quadriceps; GAS: gastrocnemius; EDL: extensor digitorum longus. Data are mean ± s.e.m. Each dot represents one mouse.


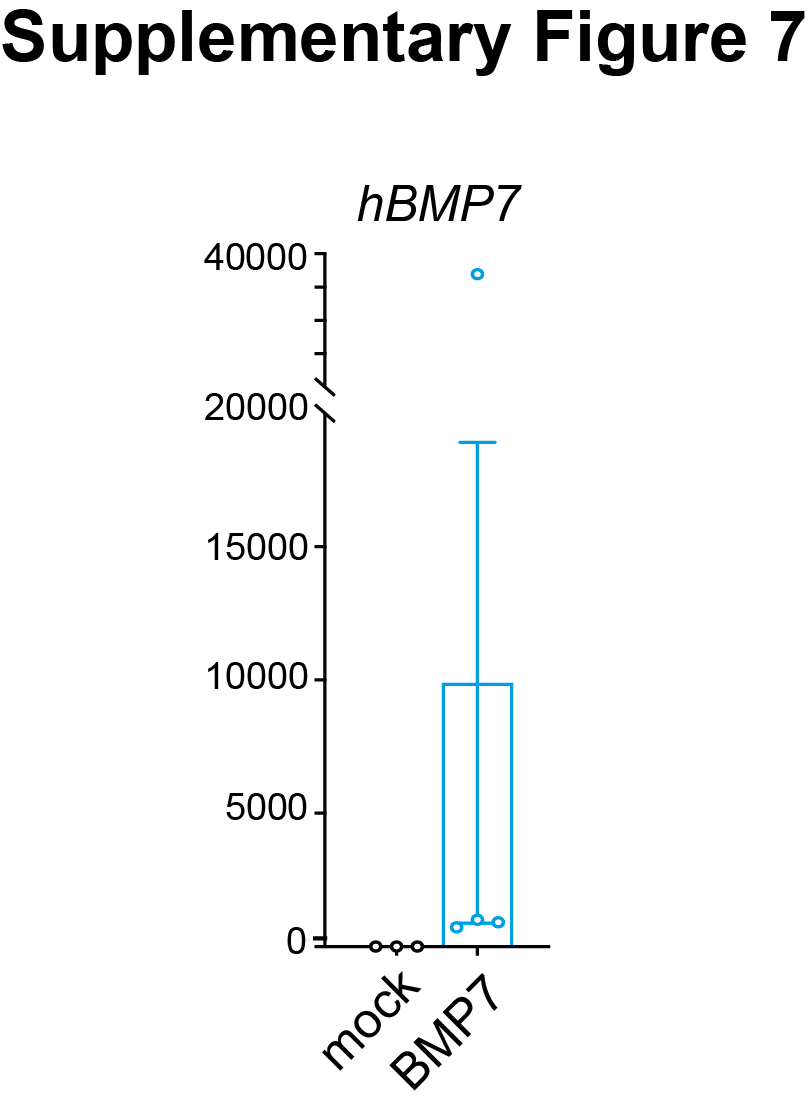


**Supplementary Figure 7.** mRNA expression levels of known human *BMP7* (*hBMP7*) normalised to *Gapdh* housekeeping gene in skeletal muscle from SBMA mice treated AAV9-BMP7-eGFP (BMP7) compared to AAV9-eGFP (mock). Data are mean ± s.e.m. Each dot represents one mouse.


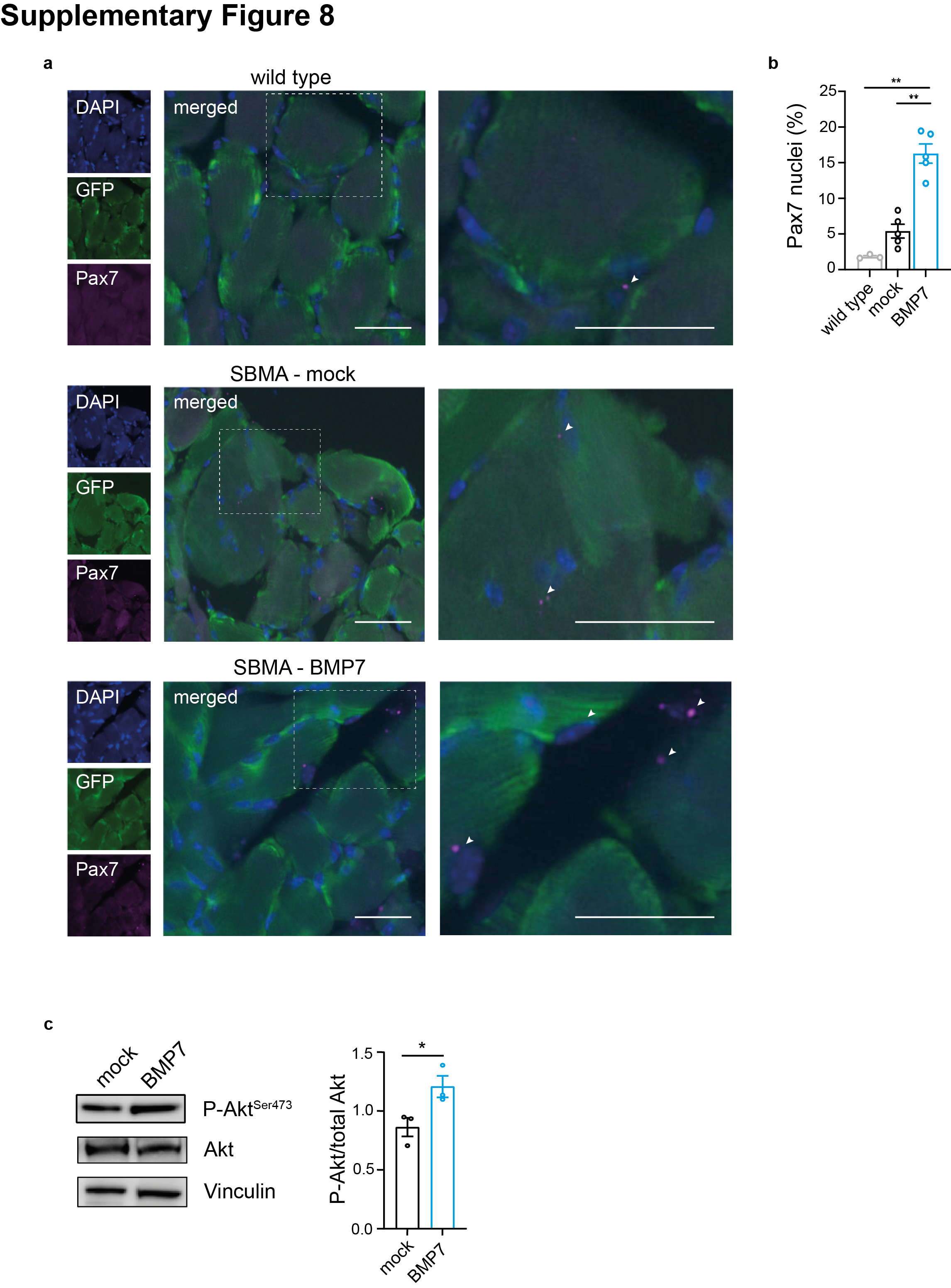


**Supplementary Figure 8. a**, Representative images of skeletal muscle from wild type and SBMA mice, treated with AAV9-eGFP (mock) or AAV9-BMP7-eGFP (BMP7), stained with Pax7 antibody (magenta) and DAPI (blue). Arrowheads indicate the Pax7 signal. Representative magnification of myofiber is shown on the side. Scale bar, 50 µm. **b**, Quantification of number of Pax7 positive cells relative to total number of nuclei. **c**, Whole cell extracts from skeletal muscle of SBMA mice were resolved by SDS PAGE followed by immunoblotting using P-Akt (Ser473), Akt, and Vinculin antibodies. Quantification of P-Akt levels relative to non-phosphorylated Akt and normalised to Vinculin is displayed on the right. Data are mean ± s.e.m. Each dot represents one mouse (*n* = 3 per group).


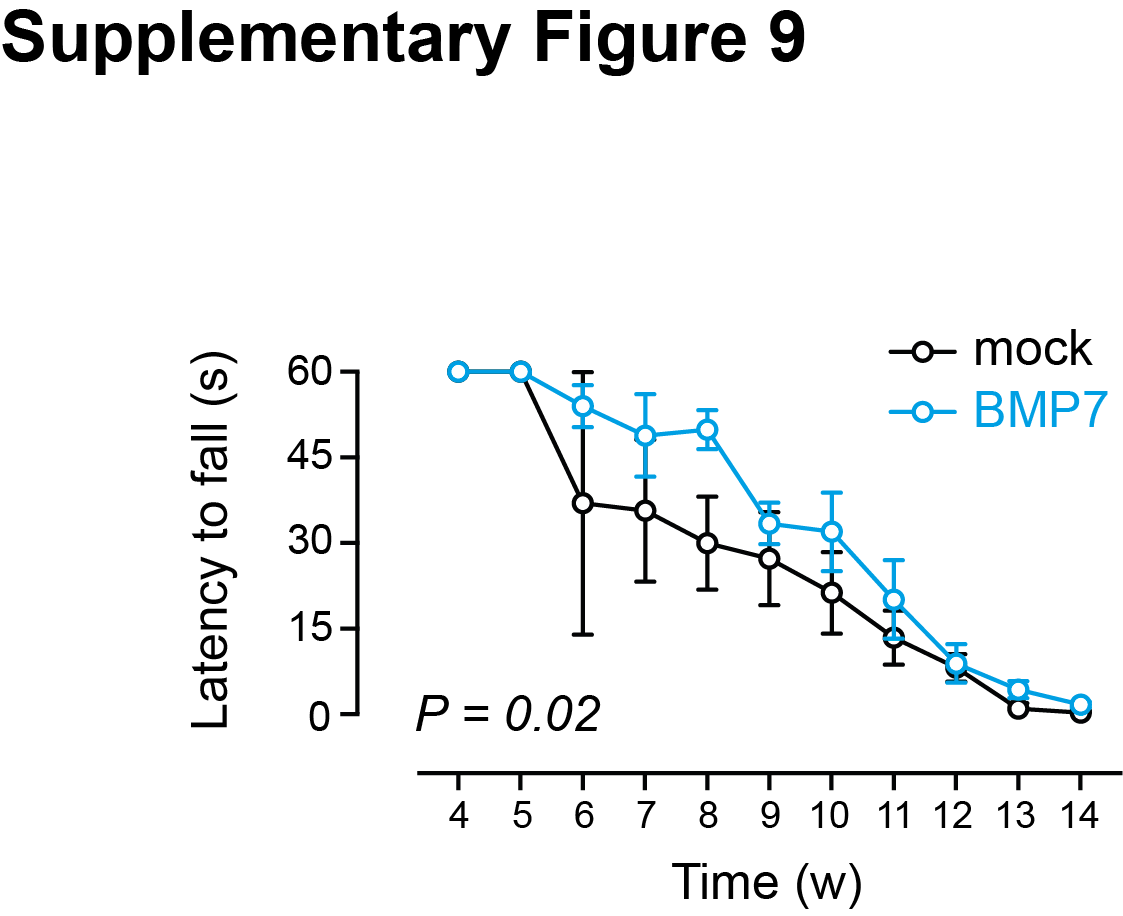


**Supplementary Figure 9.** Rotarod performances over time, expressed as mean ± s.e.m. latency to fall, of SBMA mice treated with AAV9-control and AAV9-BMP7 (two-way ANOVA) (*n* = 10 mice per group).


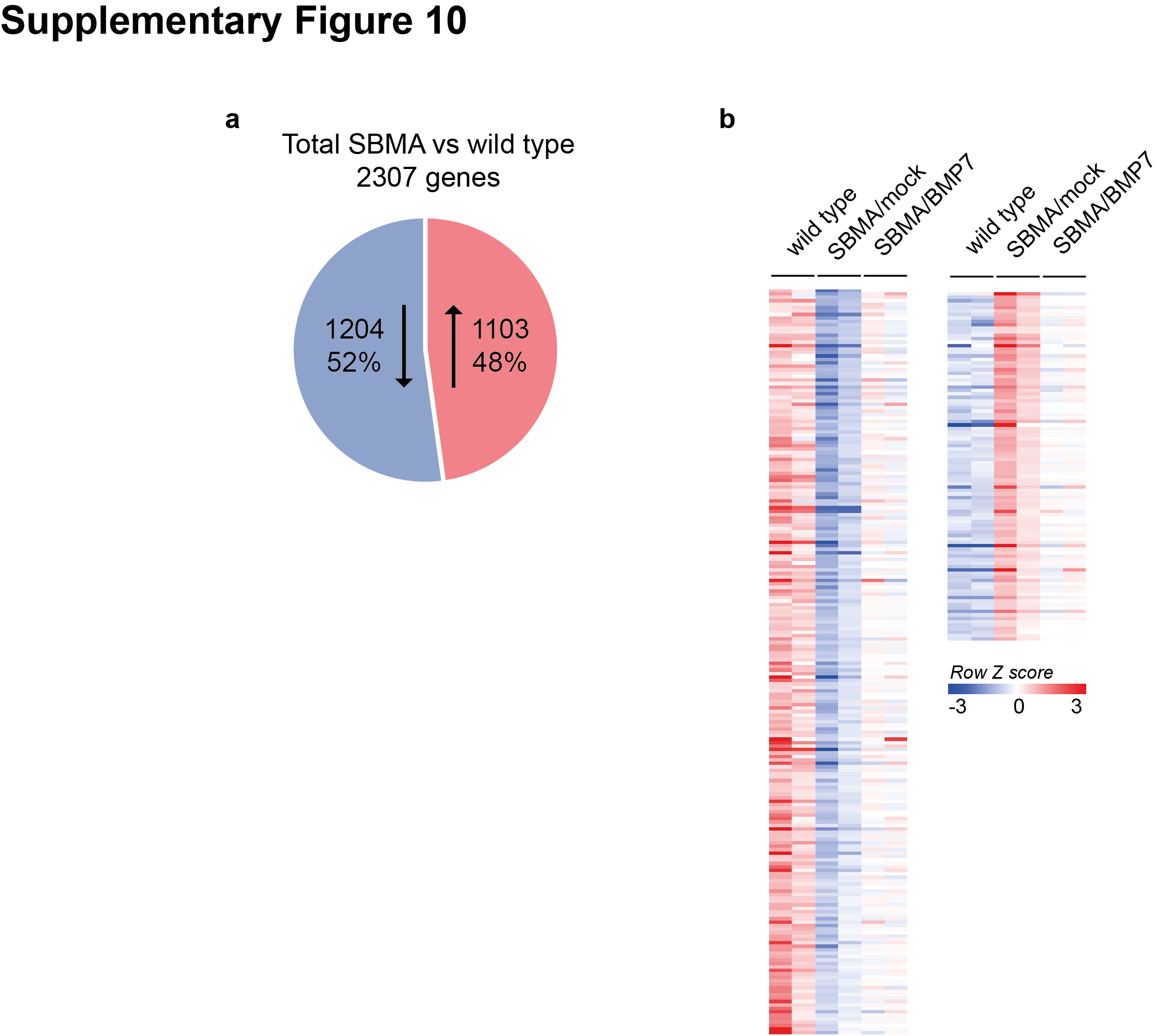


**Supplementary Figure 10.** **a**, Pie chart shows the proportion of upregulated (red) and downregulated (blue) transcripts in quadriceps muscle from SBMA mice compared to wild type mice (*n* = 2 mice per group). **b**, Heat map of z-scores for the top differentially expressed genes, showing partial restoration in skeletal muscle of BMP7-treated SBMA mice compared to mock-treated littermates.


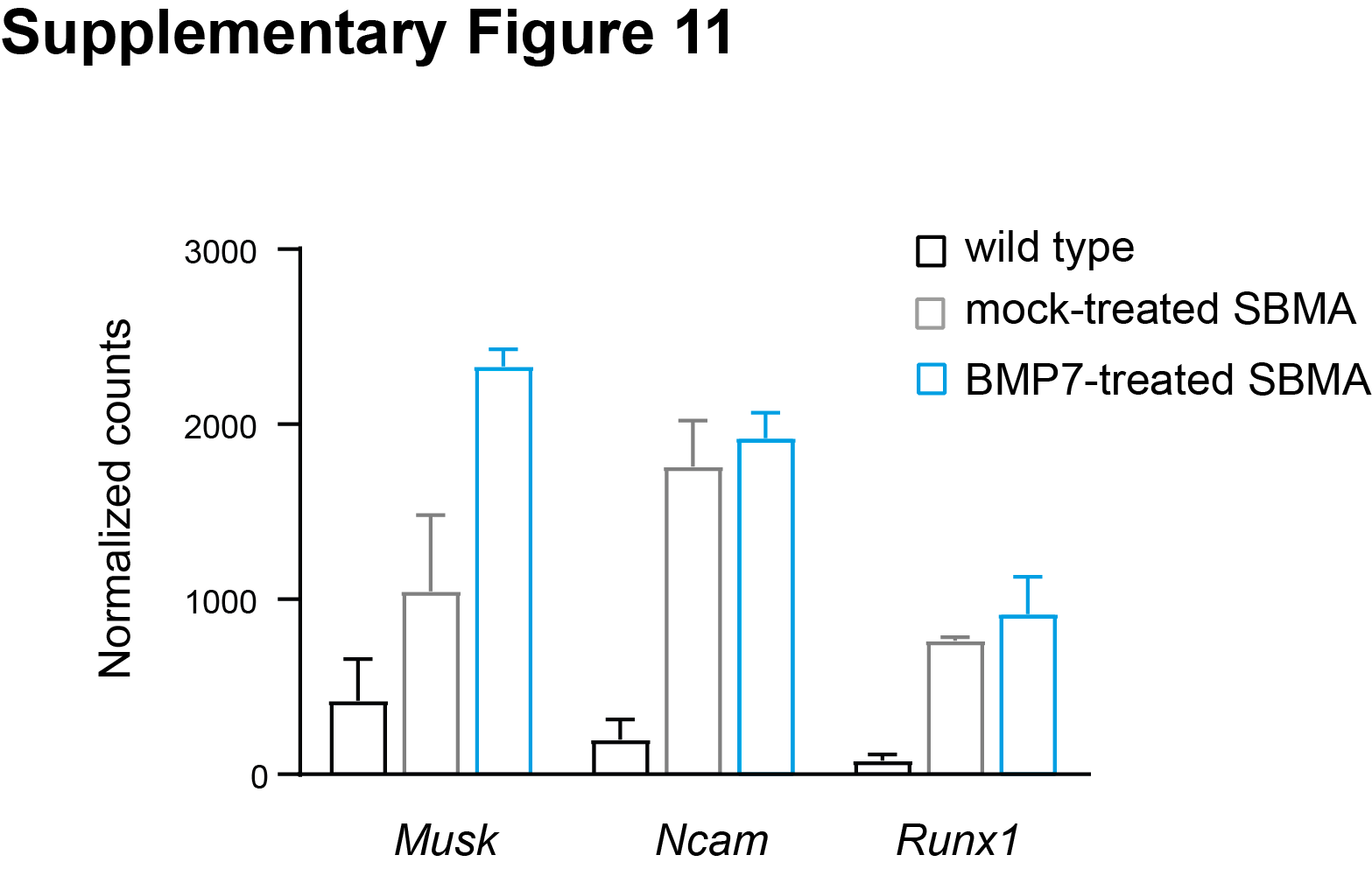


**Supplementary Figure 11**. mRNA expression levels, expressed as log-normalized counts, in gastrocnemius muscle of wild type and mock- or BMP7-treated SBMA mice of denervation-dependent transcripts (n = 2 per group).
